# HERC1 oncogene enhances stemness and tumorigenic potential in CD44^+^-derived organoids of head and neck squamous cell carcinoma through IL-6/STAT3 signaling

**DOI:** 10.1038/s41388-026-03725-9

**Published:** 2026-04-11

**Authors:** Eunjin Jeong, Hye Lin Kim, Seohee Park, Seojin Jang, Jamin Ku, Hajeong Kim, Haeun Kim, Seo Lyn Choi, Kang Pa Lee, Suji Baek, Jeong-Yoon Yang, Jung Ho Park, Jangok Yeo, Jae Jun Lee, Sei Young Lee, Seok-Hyung Kim, Hong Sook Kim, Chang-Whan Yoon, Sang-Hyuk Lee

**Affiliations:** 1 Department of Health Sciences and Technology, Samsung Advanced Institute for Health Sciences & Technology, Sungkyunkwan University, Seoul, South Korea; 2 Department of Metabiohealth, Sungkyunkwan University, Suwon, South Korea; 3 Department of Otorhinolaryngology-Head and Neck Surgery, Kangbuk Samsung Hospital, Sungkyunkwan University School of Medicine, Seoul, South Korea; 4 Medical Research Institute, Kangbuk Samsung Hospital, Sungkyunkwan University School of Medicine, Seoul, South Korea; 5 Research and Development Center, UMUST R&D corporation, Seoul, South Korea; 6 Department of Medicine, Kangbuk Samsung Hospital, Samsung Kangbuk Hospital, Sungkyunkwan University School of Medicine, Seoul, South Korea; 7 Department of Otorhinolaryngology, Eulji University Hospital, Eulji University College of Medicine, Daejeon, South Korea; 8 Preclinical Support Center, Osong Medical Innovation Foundation (KBIOHealth), Cheongju-si, South Korea; 9 Department of Otorhinolaryngology-Head and Neck Surgery, Chung-Ang University Hospital, Chung-Ang University College of Medicine, Seoul, South Korea; 10 Department of Pathology, Samsung Medical Center, Sungkyunkwan University School of Medicine, Seoul, South Korea; 11 Department of Biological Sciences, Sungkyunkwan University, Suwon, South Korea

**Keywords:** Head and neck cancer, Cancer stem cells, Cancer microenvironment

## Abstract

HECT and RCC1-like domain-containing protein 1 (HERC1), a large E3 ubiquitin ligase, has been implicated in neural development and genome stability, but its role in cancer remains unclear. This study identifies HERC1 as a critical regulator of cancer stemness, metastasis, and chemoresistance in head and neck squamous cell carcinoma (HNSCC). CD44⁺ HNSCC organoids with shRNA-mediated HERC1 knockdown were assessed for stemness, EMT, and IL-6/STAT3/HERC1 signaling using molecular assays, CAF co-culture, xenografts, and tissue immunohistochemistry. High HERC1 expression in TCGA-HNSCC datasets was associated with enrichment of stemness signatures. HERC1 knockdown in CD44⁺ cells reduced Sox2, and Slug expression, suppressed EMT, and impaired metastatic potential in Transwell assays and in vivo models. CD44⁺ cells formed organoids in a HERC1-dependent manner. CAF co-culture showed that IL-6 promoted organoid invasiveness through STAT3 activation and HERC1 upregulation. Mechanistic validation revealed that HERC1 modulation altered p-STAT3, p-ERK, CD44, and Slug levels, and STAT3 inhibition reduced HERC1 expression, defining a p-STAT3–HERC1–p-ERK axis. IL-6 neutralization or HERC1 inhibition sensitized organoids to 5-fluorouracil and cisplatin, and combined HERC1 knockdown with 5-FU markedly reduced tumor growth and increased apoptosis. Tissue arrays confirmed elevated HERC1 and pathway markers in advanced HNSCC. These findings define an p-STAT3–HERC1–p-ERK signaling axis that promotes cancer stemness and chemoresistance through CD44^+^ tumor–stromal crosstalk. Targeting HERC1 may offer a promising strategy to eliminate cancer stem-like cells in HNSCC.

## Introduction

Head and neck squamous cell carcinoma (HNSCC), a heterogeneous group of tumors, is the sixth most common cancer worldwide and is characterized by high morbidity, recurrence, and poor 5-year survival rate, especially in advanced stages [1, 2].

A major obstacle in HNSCC management is the presence of cancer stem-like cells (CSCs) that are capable of self-renewal, differentiation, and therapy resistance [3–5]. CSCs are characterized by their ability to self-renew and differentiate into heterogeneous cancer cell lineages, thereby sustaining tumorigenesis. Several markers have been proposed to identify CSCs, including CD44, ALDH1, and CD133 [6–8]. These markers are associated with aggressive tumor behavior and poor clinical outcomes. CD44, a cell surface glycoprotein, is a well-established marker of CSCs in HNSCC and is correlated with poor prognosis and metastasis [9]. CD44^high^ cells in HNSCC have enhanced tumorigenic potential and resistance to conventional therapies, emphasizing the need for CSC-targeted interventions [10–12].

The tumor microenvironment (TME), epithelial-mesenchymal transition (EMT), and signaling pathways such as Wnt, Notch, and Hedgehog contribute to the maintenance of the CSC phenotype in head and neck malignancies [13]. Recent studies have highlighted the importance of tumor-stromal interactions in sustaining CSC phenotypes. Cancer-associated fibroblasts (CAFs) secrete pro-inflammatory cytokines such as Interleukin-6 (IL-6), which activate STAT3 (Signal transducer and activator of transcription 3) signaling and contribute to tumor progression [14–16]. The IL-6 signaling pathway is one of the most dysregulated pathways in cancer. Fibroblasts, especially in their activated form as CAFs, are key components of the tumor niche. These cells secrete a variety of growth factors (e.g., TGF-β, EGF), extracellular matrix (ECM) components, and cytokines that promote EMT, angiogenesis, and CSC maintenance in HNSCC [16–19]. CAFs contribute to the physical restructuring of the tumor stroma and create a supportive niche that protects tumor cells from immune surveillance and enhances their metastatic potential. CAFs directly interact with HNSCC CSCs, facilitating their self-renewal and resistance to chemotherapy and radiotherapy through paracrine signaling and ECM remodeling [20–25]. This dynamic interaction between CD44^+^ CSCs and CAFs suggests that disrupting their crosstalk may improve therapeutic outcomes and reduce tumor recurrence. However, downstream effectors that mediate these microenvironmental cues remain poorly understood.

HECT and RCC1-like domain-containing protein 1 (HERC1) is a large E3 ubiquitin ligase implicated in vesicle trafficking, neural development, and genome stability [26]. HERC1 may act as a tumor suppressor or regulator of oncogenic pathways, depending on the cellular context [27, 28]. Alterations in HERC1 expression have been observed in certain malignancies, including glioblastoma, breast cancer, and colorectal cancer, but the underlying mechanisms remain elusive [28–31]. Although emerging evidence links HERC family members to tumorigenesis, the role of HERC1 in cancer stemness, metastasis, and drug resistance has not yet been elucidated.

## Materials and methods

### Cell lines and reagents

Human HNSCC cell lines SCC-15, SCC-25, and QLL-1, WS1 (human fibroblasts) were obtained from the American Type Culture Collection (ATCC) and cultured in Dulbecco’s modified Eagle’s medium (DMEM) supplemented with 10% fetal bovine serum (FBS), 1% penicillin-streptomycin, and 1% glutamine. The cells were maintained at 37 °C in a humidified incubator with 5% CO₂.

5-Fluorouracil (5-FU; Cat. F6627), Cisplatin (Cat. P4394), and Puromycin (Cat. P9620) were purchased from Sigma-Aldrich (St. Louis, MO, USA). U0126 (ERK inhibitor; Cat. No. S1102) and WP1066 (STAT3 inhibitor; Cat. No. HY-15312) were purchased from MedChemExpress (MCE, USA).

### Lentiviral transduction and HERC1 knockdown

Stable knockdown of HERC1 was achieved using a short hairpin RNA (shRNA) construct targeting HERC1 (sh.HERC1; sequence: CAGATTGTTGAGCGCTTATTT; GACTGCTTTATGACCATATTA; GAGCGCAAGCCATGATCTATA; Vector Builder, USA) with scrambled shRNA as a control (sh.Scr). Transfection was performed using Lipofectamine™ 3000 reagent (Cat. L3000015, Invitrogen) following the manufacturer’s protocol. After transfection, the cells were selected using puromycin (5 μg/mL; Sigma-Aldrich) for 7 days. Knockdown efficiency was validated by Western blotting analysis.

### Cell growth and proliferation

For cell counting, 1 × 10^3^ cells transduced with sh.HERC1 were seeded in 6-well plates. Cells were harvested and counted by trypan blue exclusion at 24, 48, and 72 h. The growth was plotted over time.

For the proliferation assay, 5 × 10^3^ cells/well were seeded in 96-well plates. After 24, 48, and 72 h, the WST-8 reagent (Cat. WQ3000, WST-8 Cell Viability Assay Kit) was added to each well following the manufacturer’s instructions. Absorbance was measured at 450 nm using a microplate reader and the data were analyzed accordingly.

### Western blotting analysis

Total protein was extracted using RIPA buffer (sc-24948, Santa Cruz) containing protease and phosphatase inhibitors (Cat. 5872, Cell Signaling). Protein lysates (20 µg) were resolved by SDS-PAGE and transferred to nitrocellulose membranes. Membranes were incubated with primary antibodies against HERC1 (Cat. Sc-393950, Santa Cruz), CD44 (Cat. A1351, Abclonal; Cat. 3570, Cell Signaling), Nanog (Cat. A22625, Abclonal), Sox-2 (Cat. 23064, Cell Signaling), Oct4 (Cat. A7920, Abclonal), c-Myc (Cat. sc-42, Santa Cruz; Cat. AB32072, Abcam), Slug (Cat. 9585, Cell Signaling), Snail (Cat. 5243, Abclonal), Zeb1 (Cat. A5600, Abclonal), N-cadherin (Cat. 1MA1-159, Invitrogen), E-cadherin (Cat. AB15148, Abcam), Vimentin (Cat. MA3-745, Invitrogen; Cat. AB92547, Abcam), p-STAT3 (Y705) (Cat. sc-8059, Santa Cruz; Cat. 9145, Cell Signaling), STAT3 (Cat. ab68153, abcam), cleaved Caspase-3 (Cat. 9661, Cell Signaling), Bcl-2 (Cat. 13-8800, Invitrogen), p44/42 MAPK (Erk1/2) (Cat. 4695, Cell Signaling), ERK1 (sc-376852, Santa Cruz), Phospho-SAPK/JNK (Thr183/Tyr185) (Cat. 4668, Cell Signaling), JNK1 (sc-1648, Santa Cruz), Phospho-p38 MAPK (Thr180/Tyr182) (Cat. 4511, Cell Signaling), p38 MAPK (Cat. 9212, Cell Signaling), and β-actin (Cat. MAB8929, R&D system) overnight at 4 °C. After incubation with horseradish peroxidase-conjugated secondary antibodies, the bands were visualized using ECL substrate (Cat. LF-QC01060, AbFrontier).

### Magnetic cell sorting (MACS)

CD44^+^ cells were isolated from the dissociated tumor spheroids using anti-CD44 MicroBeads (Cat. 130-095-194, Miltenyi Biotec) following the manufacturer’s instructions. Briefly, CD44^+^ cells (spheroids) were enzymatically dissociated into single-cell suspensions using Accutase (cat. A1110501; Life Technologies), and incubated with anti-CD44 magnetic beads at 4 °C for 15 min. The labeled cells were then passed through MS columns placed in a magnetic field (cat. 130-042-201, Miltenyi Biotec), and CD44⁺ cells were collected from the column. The purity of the isolated population was assessed by flow cytometry using a fluorophore-conjugated anti-CD44 antibody, and only samples with >90% CD44⁺ purity were used for downstream analysis.

### Spheroid and organoid formation assays

For spheroid assays, 1 × 10⁴ cells were seeded in spheroid medium in low-attachment plates and cultured for up to 3–5 days with Advanced DMEM/F12, B27 (1X; Cat. 17504044, Gibco), N2 (1X), EGF (10 ng/mL; Cat. 17502048, Gibco), and bFGF (10 ng/mL; Cat. GF003AF, Sigma Aldrich) as spheroid culture conditions.

For organoid formation [32, 33], CD44⁺ cells were isolated by Magnetic-Activated Cell Sorting (MACS; Miltenyi Biotec) and overlaid with DMEM/F12 supplemented with penicillin/streptomycin, 10 mmol/L HEPES, GlutaMAX, 1X B27 (Cat. 17504044, Gibco), 1X N2 (Cat. 17502048, Gibco), 1.25 mmol/L N-acetylcysteine (Cat. A9165, Sigma-Aldrich), 0.05 mg/mL Epidermal Growth Factor (EGF; Cat. 9644, Sigma Aldrich), 0.1 mg/mL Fibroblast Growth Factor-basic (FGF-basic; Cat. GF003AF, Sigma Aldrich), 0.01 mmol/L gastrin I (Cat. G9145, Sigma Aldrich), 10 mmol/L Nicotinamide (Cat. N0636, Sigma Aldrich), 10 mmol/L Y-27632 (Cat. Y0503, Sigma Aldrich), and SB202190 (Cat. S7067, Sigma Aldrich); 1 mmol/L Prostaglandin E2 (Cat. 2296, Tocris Bioscience, Tocris Bioscience); 0.1 mg/mL Fibroblast Growth Factor-10 (FGF-10; Cat. 100-26, PeproTech); 0.5 mmol/L A83-01 (Cat. 2939, R&D Systems), and L-WRN Conditioned Media (Wnt3A, Rspondin 1, mNoggin; SCM105, Sigma) in 24-well plates. The organoid culture medium was completely refreshed every 3–4 days. Organoids were passaged every week at a 1:3–1:6 split ratio by removing them from Matrigel using BD Cell Recovery Solution (Cat. 354253, BD Biosciences) following the manufacturer’s instructions and transferring them to fresh Matrigel (Cat. 356255, BD Biosciences). Organoid numbers and sizes were quantified using the ImageJ software (NIH) [34].

### Immunocytochemistry

WS1 fibroblasts and spheroids were fixed with 4% paraformaldehyde for 15 min, permeabilized with 0.1% Triton X-100 in PBS for 10 min. and blocked with 1% BSA in PBS for 1 h. Cells were incubated overnight at 4 °C with CD44 (FITC-conjugated; Cat. 130-113-334, Miltenyi Biotec), Sox-2 (Cat. 23064, cell Signaling), Slug (Cat. 9585, Cell Signaling), or FAP (Cat. sc-65398, Santa Cruz) antibodies, followed by Alexa Fluor 488/594/647–conjugated secondary antibodies (Cat. A-11008; Cat. A32744; Cat. A32728TR, Life Technologies). Nuclei were counterstained with DAPI (Cat. F6057, Sigma), and images were acquired using a Zeiss confocal microscope and processed with Imaris 7.6.

### Migration and invasion assays

Cell migration and invasion were assessed using Transwell assays with Boyden chambers (8 µm pore size; Corning). For invasion assays, the upper surfaces of the inserts were coated with Matrigel (Cat. 354234), whereas the migration assays were performed without Matrigel. A total of 2 × 10⁴ cells were suspended in serum-free medium and seeded into the upper chamber. The lower chamber was filled with medium containing 10% FBS or fibroblasts as chemo-attractants. After 24–72 h of incubation at 37 °C, non-migrated or non-invaded cells were removed from the upper surface of the membrane using a cotton swab. Cells that had migrated or invaded the lower surface were fixed and stained using a staining kit (Cat. 898125, Diff Quick stain set) and counted in five randomly selected fields per insert under a light microscope.

### Drug sensitivity and apoptosis assays

To assess chemotherapeutic sensitivity, CD44⁺ organoids were treated with increasing concentrations of 5-fluorouracil (5-FU; 0.01–100 µM; Cat. F6627, Sigma-Aldrich) or cisplatin (0.01–100 µM; Cat. P4394, Sigma-Aldrich) for 24–72 h. Drug treatments were performed in an organoid culture medium under standard conditions. Cell viability was measured using a WST-8 Cell Viability Assay Kit (Cat. WQ3000, UMUST R&D Co.) following the manufacturer’s instructions. Absorbance was recorded at 450 nm using a microplate reader, and dose-response curves were generated to calculate IC₅₀ values.

Apoptosis was assessed by IHC/IF staining of cleaved Caspase-3 in paraffin-embedded organoid sections, and western blotting analysis of cleaved Caspase-3 and Bcl-2 protein levels. For IHC and IF, organoids were fixed in 4% paraformaldehyde, embedded in paraffin, sectioned, and stained with an anti-cleaved Caspase-3 antibody (Cat. 9664, 1:200 dilution, Cell Signaling), and an anti-Bcl-2 antibody (Cat. 13-8800, Invitrogen). For Western blotting, total protein was extracted using RIPA buffer (sc-24948, Santa Cruz) supplemented with protease and phosphatase inhibitors (Cat. 5872, Cell Signaling) and analyzed by Western blotting.

### Immunohistochemistry (IHC) and immunofluorescence (IF)

Formalin-fixed, paraffin-embedded xenograft and lung metastasis tissues were sectioned (3 μm), deparaffinized, rehydrated, and subjected to heat-induced antigen retrieval in citrate buffer (pH 6.0; Cat. 005000, Invitrogen). After blocking with 1% BSA, sections were incubated overnight at 4 °C with primary antibodies against HERC1 (Cat. PA5-62033, Thermo Scientific), phospho-STAT3 (Y705) (Cat. 9145, Cell Signaling), CD44 (Cat. 3570, Cell Signaling), Sox-2 (Cat. 14962, Cell Signaling), Ki-67 (Cat. 9449, Cell Signaling), cleaved Caspase-3 (Cat. 9661, Cell Signaling) or pan-Cytokeratin (Cat. 67306, Cell Signaling).

For IHC, HRP-conjugated secondary antibodies were applied, visualized with DAB (Cat. SK-4100, Vector Labs.), and counterstained with hematoxylin.

For IF, Alexa Fluor 488/594/647–conjugated secondary were used (Cat. A-11008; Cat. A32744; Cat. A32728TR, Life Technologies), nuclei were counterstained and mounted with DAPI (Cat. F6057, Sigma). Images were captured using a Zeiss confocal microscope under identical settings, and quantitative analysis was performed on ≥ 5 random high-power fields per sample using Imaris 7.6 or ImageJ.

### Mouse xenograft and metastasis assays

All animal experiments were conducted in accordance with protocols approved by the Institutional Animal Care and Use Committee (IACUC) of Kangbuk Samsung Hospital (25-144-A1-N). For subcutaneous xenograft assays, 5 × 10⁶ sh.Scr or sh.HERC1 cells suspended in 100 μL of PBS:Matrigel (1:5) were injected into the flanks of 6-week-old NOD/SCID mice. Tumor growth was monitored every 3 days using calipers, and tumor volume was calculated using the following formula: (length × width²)/2.

For experimental metastasis assays, 1 × 10⁶ CD44⁺-derived organoids were resuspended in sterile PBS and injected into the lateral tail vein of NOD/SCID mice. After 3 weeks, the mice were euthanized and the lungs were harvested, fixed in 10% neutral-buffered formalin, embedded in paraffin, and sectioned for hematoxylin and eosin (H&E) staining, immunohistochemistry (IHC), and immunofluorescence (IF) to assess metastatic burden.

### Co-culture system with fibroblasts

Human fibroblasts (WS-1) were cultured in DMEM supplemented with 10% FBS. For indirect co-culture experiments, 2 × 10⁴ fibroblasts per well were seeded into the lower chamber of a Transwell system (0.4 μm pore size), while CD44⁺ cells were seeded in the upper chamber. For three-dimensional (3D) direct co-culture, 2 × 10⁴ fibroblasts and 5 × 10^3^ CD44⁺ organoids (4:1) were embedded together in Matrigel domes and cultured for 7 days. Organoid morphology and invasive behavior were assessed using bright-field microscopy.

### Enzyme-linked immunosorbent assay (ELISA)

IL-6 concentration in conditioned media (CM) was measured using a human IL-6 Quantikine ELISA kit (R&D Systems, Cat. D6050), following the manufacturer’s protocol. CM was collected from fibroblasts, cancer cell lines, and CD44⁺ cells cultured individually or in co-culture systems (indirect Transwell or 3D Matrigel) for 48–72 h. Before collecting, CM was centrifuged at 300 × *g* for 5 min to remove cellular debris and stored at −80 °C until analysis. Absorbance was measured at 450 nm (with correction at 570 nm) using a microplate reader (SPARK, TECAN KOEA), and IL-6 concentrations were calculated using a standard curve generated with recombinant human IL-6 provided in the kit.

### Cytokine treatment and blocking experiments

To modulate IL-6 signaling, CD44⁺ cells and organoids were treated with recombinant human IL-6 (20 ng/mL; R&D Systems, Cat. 206-IL) or an anti-IL-6 neutralizing antibody (1 µg/mL; Cat. MAB206, R&D Systems) for 72 h. Treatments were performed in a low-serum (0.5% FBS) culture medium to minimize background cytokine activity. For blocking experiments, cells were pre-incubated with low serum concentration for 2 h before IL-6 stimulation. After 72 h, cells and organoids were harvested for protein extraction or fixed for IF analysis. Western blotting and IF were used to evaluate the downstream signal activation and target protein expression, respectively.

### Human tumor microarray (TMA) analysis

Commercial tissue microarray (TMA) slides (Cat. HN802d, TissueArray) containing HNSCC tissues at various stages and normal tissue controls were stained with antibodies against HERC1, CD44, and fibroblast activation protein (FAP). IHC was performed using standard protocols with diaminobenzidine as the chromogen. For each sample, five representative fields were scored for marker expression. Stained slides were digitally scanned using a whole-slide scanner (Pannoramic 150 Digital Scanner) at 20× and 40× magnifications to generate high-resolution images.

Image analysis was conducted using ImageJ software (NIH), allowing quantification of staining intensity, positive area, and cell counts. For IF staining, nuclei were counterstained with DAPI and used as a reference for cell segmentation and co-localization analysis. Quantitative data were derived from three independent experiments and presented as mean ± standard deviation (SD).

### Data acquisition and processing

TCGA-HNSC bulk RNA-seq were downloaded using the R package TCGAbiolinks. Specifically, the GDCquery() and GDCdownload() functions were used to retrieve HTSeq-Counts from The Cancer Genome Atlas (TCGA) Head and Neck Squamous Cell Carcinoma (HNSC) cohort. Clinical metadata, including patient survival information, were retrieved from cBioPortal(http://www.cbioportal.org) for consistency and completeness.

Single-cell RNA-seq data from primary and metastatic HNSC tissues (GSE181919) were obtained from the Gene Expression Omnibus (GEO). The UMI count matrix and corresponding barcode-level metadata were loaded into R and preprocessed using the Seurat package (v4.3.0). Cell type annotation was based on the original metadata, and fibroblasts were subset for downstream analysis. For IL-6 expression analysis, fibroblasts from NL (normal adjacent tissue) and LP (leukoplakia) tissues were considered as normal fibroblasts, while those from CA (primary tumor) and LN (lymph node metastasis) tissues were classified as cancer-associated fibroblasts (CAFs).

### Expression-based stratification

For CD44-based analysis, TCGA-HNSC patients were divided into high and low groups using the median expression level of CD44 as the cutoff. For combined CD44 and HERC1 analysis, the expression values of CD44 and HERC1 were first averaged within each patient, and the overall mean of these per-patient averages was used to define the cutoff. Patients with values above this mean were categorized as high, and those below as low.

### Survival analysis

Overall survival analysis was performed using clinical metadata from the cBioPortal TCGA-HNSC cohort, which includes survival time (in months) and vital status (alive or deceased). Patients were grouped based on gene expression levels, using the same stratification strategy described above (combined CD44/HERC1 average). Kaplan–Meier survival curves were generated using the survival and survminer R packages. Samples with missing survival or expression data were excluded from the analysis.

### Differential expression and gene set enrichment analysis

Differential gene expression analysis between high and low groups was performed using the limma-voom pipeline. Genes with a B-statistic greater than 5 were considered significantly differentially expressed and were used to construct ranked gene lists for gene set enrichment analysis (GSEA). GSEA was conducted using the clusterProfiler R package (v4.6.2) with Gene Ontology Biological Process (GO:BP) as the reference gene set collection. Genes were ranked by their B-statistic, and enriched terms with a false discovery rate (FDR) < 0.05 were considered statistically significant. Enrichment plots were visualized using gseaplot2() or custom ggplot2-based visualizations.

### Statistical analysis

Data are presented as mean ± standard deviation (SD). All experiments were performed in triplicates. Statistical significance was determined using Student’s *t*-test or one-way ANOVA followed by Tukey’s post hoc test, or Wilcoxon rank-sum test for non-parametric comparisons. A *P* < 0.05 was considered statistically significant. GraphPad Prism 9, ImageJ, Microsoft Excel, and ggplot() in R were used for all the analyses.

## Results

### Elevated HERC1 expression in HNSCC correlates with CD44 and suggests clinical relevance

**Fig. 1:**
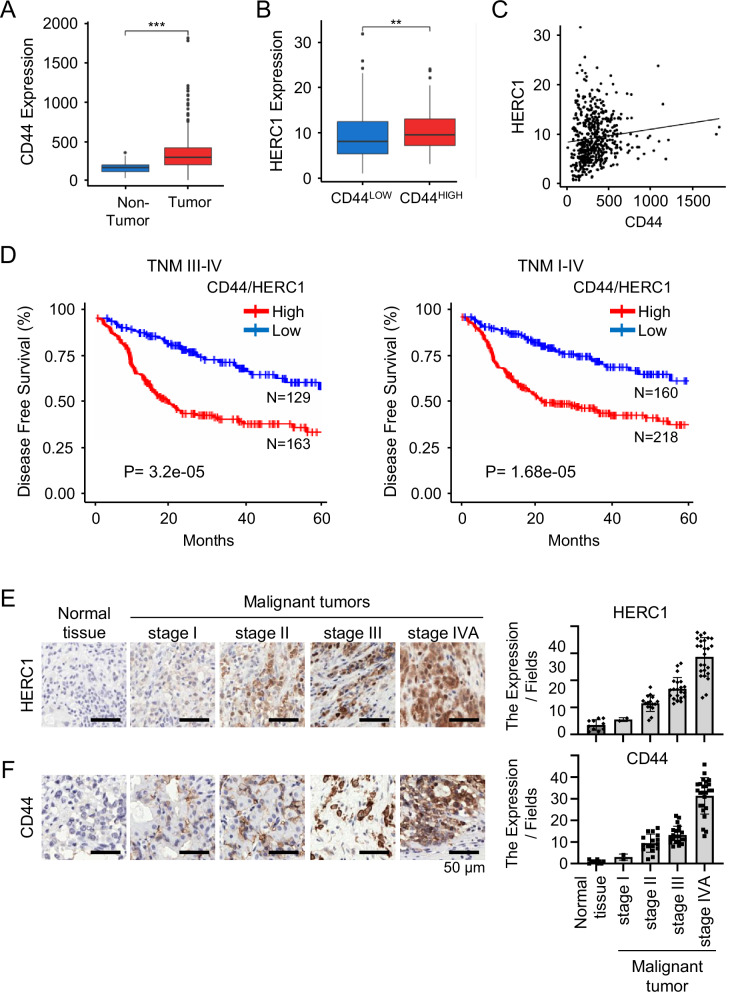
HERC1 expression is elevated in HNSCC and correlates with CD44 expression. **A** Box plots showing significantly higher CD44 transcript levels in HNSCC tumor tissues compared to non-tumor tissues in TCGA-HNSC dataset (**p < 0.001). **B** Comparison of HERC1 expression in CD44^High^ vs. CD44^Low^ tumor samples shows significantly higher expression in the CD44-high group (*p < 0.01). **C** Scatter plot demonstrating a positive correlation between CD44 and HERC1 expression (*R* = 0.11, *p* = 0.0094). **D** Kaplan–Meier survival curves stratified by CD44 and HERC1 co-expression levels. Patients with high CD44^High^/HERC1^High^ expression show a trend toward poorer overall survival (TNM III–IV; *p* = 3.2e-05, TNM I–IV; *p* = 1.68e-05). **E**, **F** Representative images from human tumor microarray (TMA) slides showing HERC1 and CD44 expression in normal and malignant head and neck tissue samples.

To explore the clinical relevance of HERC1 in head and neck squamous cell carcinoma (HNSCC), we analyzed The Cancer Genome Atlas (TCGA-HNSC) dataset. Transcript-level analysis revealed that CD44 expression was significantly elevated in HNSCC tumor tissues compared to adjacent non-tumor tissues (Fig. 1A). Stratification by CD44 expression, a marker of cancer stem-like cells, further demonstrated that HERC1 levels were significantly higher in CD44-high tumors (Fig. 1B), suggesting a potential association between HERC1 and cancer stemness. Pearson correlation analysis confirmed a positive correlation between CD44 and HERC1 expression (*R* = 0.11, *p* = 0.0094), indicating transcriptional co-regulation or functional linkage between these two factors (Fig. 1C). Kaplan–Meier survival analysis demonstrated a stage-dependent prognostic pattern. In early-stage disease (TNM I–II; Supplementary Fig. S1A), HERC1^High^/CD44^High^ expression did not significantly alter survival outcomes. However, in advanced disease (TNM III–IV; Fig. 1D), HERC1^High^/CD44^High^ expression was associated with significantly poorer overall survival. Notably, patients with TNM IV disease and HERC1^High^/CD44^High^ expression exhibited the most pronounced reduction in survival probability (Supplementary Fig. S1A). When all stages were analyzed collectively (TNM I–IV: Fig. 1D), HERC1 remained a strong negative prognostic indicator, with high-expression tumors showing significantly reduced overall survival relative to low-expression tumors. Together, these findings indicate that HERC1^High^/CD44^High^ expression becomes prognostically relevant as HNSCC progresses and may serve as a biomarker of aggressive, advanced-stage disease enriched for HERC1^+^/CD44⁺ cancer stem-like features.

Next, we examined the expression of HERC1 and CD44 in commercially available human tumor microarray (TMA) slides spanning various stages of head and neck cancer (Supplementary Fig. S1B, S1C). IHC staining revealed elevated HERC1 expression in high-grade tumor samples, with a positive correlation between HERC1 levels and tumor stage (Fig. 1E). Similarly, The CSC marker CD44 were significantly upregulated in more advanced tumors (Fig. 1F). Collectively, these data suggest that HERC1 plays a previously unrecognized role in maintaining stem cell-like phenotypes and poor prognosis in HNSCC, prompting further functional investigations.

### Establishment of HERC1^high^/CD44^+^-derived organoids in HNSCC

To investigate the role of HERC1 in regulating cancer stemness, we first performed gene set enrichment analysis (GSEA) using TCGA HNSCC datasets (Supplementary Fig. S2A). High HERC1/CD44 expression was positively correlated with cancer stem cell–associated gene signatures, suggesting a potential link between HERC1 and stem-like phenotypes. And then, Stable HNSCC cell models were established using three validated shRNA constructs targeting HERC1 (sh.HERC1). Western blotting confirmed the efficiency of HERC1 silencing (Supplementary Fig. S2B). Notably, HERC1 depletion had no significant effect on the overall cell proliferation under standard monolayer conditions, indicating that HERC1 is dispensable for general cell growth (Supplementary Fig. S2C).

**Fig. 2:**
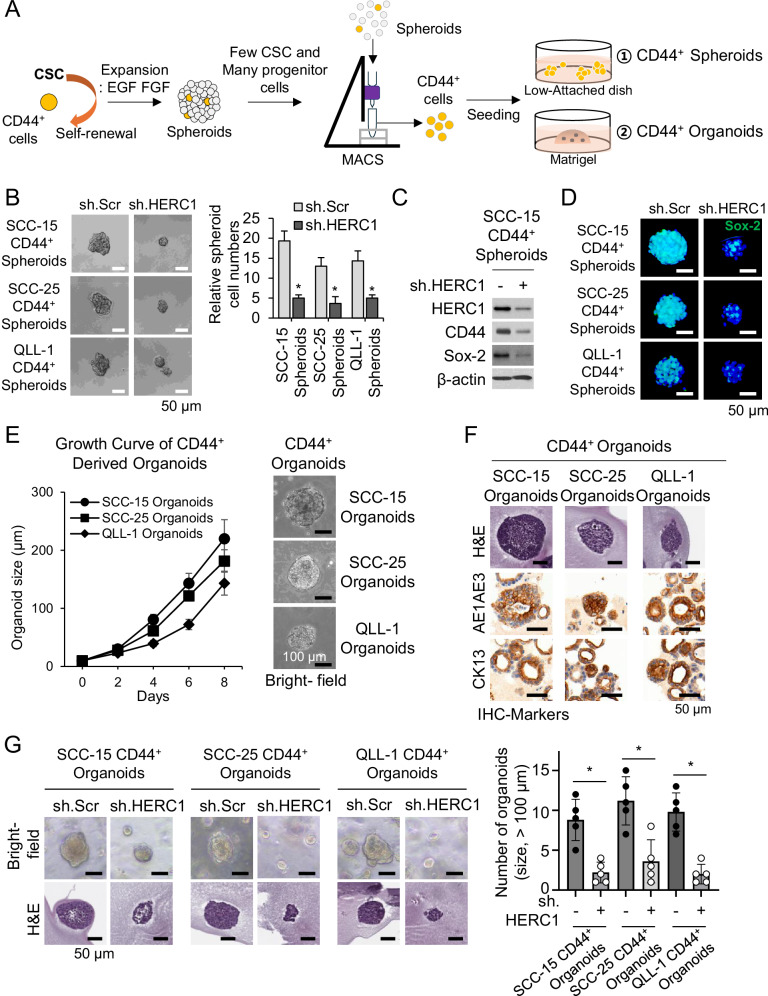
HERC1 regulates CD44⁺ cancer stem-like cell properties and organoid formation in head and neck squamous cell carcinoma (HNSCC). **A** Schematic establishment of the experimental workflow for enrichment of CD44⁺ cells from HNSCC tumors ①, and generation of CD44⁺-derived organoids ②. **B** Bright-field images and quantification of spheroid formation by CD44⁺ cells transduced with control or HERC1 sh.RNA in SCC-15, SCC-25 and QLL-1 cell lines. Scale bars, 50 μm. **C** Western blot analysis confirming HERC1 knockdown efficiency in CD44⁺ cells. **D** Immunofluorescence staining of Sox-2 (green) in CD44⁺ cells shows decreased expression upon HERC1 knockdown. Nuclei were counterstained with DAPI (blue). Scale bars, 50 μm. **E** Growth curves and representative bright-field images of CD44⁺-derived organoids with or without HERC1 knockdown. Scale bars, 100 μm. **F** Representative bright-field and hematoxylin and eosin (H&E) staining images of organoids derived from three independent HNSCC cell lines, demonstrating structural and histological features. Immunohistochemical staining shows expression of epithelial markers AE1/AE3 and CK13 in CD44⁺-derived organoids. Scale bars, 50 μm. **G** Bright-field and H&E staining images of CD44⁺-derived organoids transduced with sh.HERC1, along with quantification of organoid formation efficiency. Scale bars, 50 μm.

Spheroid-forming assays were performed under spheroid culture conditions (Supplementary Fig. S2D). We found that the spheroids were well-established. Next, we assessed the effect of HERC1 on spheroid formation under spheroid culture conditions. To assess the functional capacity of CD44⁺ stem-like cells, we isolated CD44⁺ cells from spheroids using magnetic-activated cell sorting (MACS), expanded them under spheroid culture conditions (Cancer Stem cell; Fig. 2A-①), and subsequently embedded them in Matrigel to generate organoids (Fig. 2A-②).

We next compared protein expression profiles between CD44⁺ and CD44⁻ sorted populations. Western blot analysis revealed that CD44⁺ cells expressed markedly higher levels of self-renewal–associated transcription factors (SOX-2, OCT-4, Nanog, and c-Myc) compared with CD44⁻ cells (Supplementary Fig. S2E), consistent with an enrichment of stem-like properties in the CD44⁺ fraction.

CD44⁺ cells generated abundant spheroids in the sh.Scr group, whereas HERC1 knockdown markedly reduced spheroid formation by ~60–80%, indicating impaired self-renewal capacity (Fig. 2B). Consistent with this functional decline, the stemness-related gene Sox2 was reduced in sh.HERC1 spheroids by Western blot analysis (Fig. 2C), and immunofluorescence further confirmed decreased Sox2 expression accompanied by smaller and disorganized spheroid architecture (Fig. 2D).

We first established CD44^+^-derived organoids from HNSCC cells. Isolated CD44^+^ cells with sh.HERC1 were seeded in Matrigel (Fig.2A-②). CD44⁺ cells readily formed organoids in control conditions, validating their stem-like differentiation potential (Fig. 2E and Supplementary Fig. S2F). Organoids derived from CD44⁺ cells expressed typical epithelial markers such as AE1/AE3 and CK13, confirming their tumor epithelial identity (Fig. 2F). Importantly, HERC1 knockdown significantly impaired organoid growth, resulting in a 60–80% reduction in organoid size and number compared to controls (Fig. 2G). Immunofluorescence analysis further confirmed these findings, showing strong CD44 expression localized on the surface of control organoids, whereas HERC1-depleted organoids exhibited markedly reduced CD44 and HERC1 staining intensity and disrupted organoid architecture (Supplementary Fig. S2G). To assess whether HERC1 promotes stemness in CD44⁻ cells, we ectopically expressed HERC1 in the CD44⁻ population. HERC1 overexpression increased CD44 protein levels, as confirmed by Western blot analysis (Supplementary Fig. S2H), and significantly enhanced spheroid formation compared with vector controls, indicating that HERC1 is sufficient to confer stem-like, self-renewal capacity and drive a CD44⁻ population toward a CD44⁺-like phenotype (Supplementary Fig. S2I). These findings suggest that HERC1 is essential for maintaining the self-renewal capacity of CSCs in head and neck cancer, and for supporting the generation of organoids from CD44⁺ cells.

### HERC1 promotes EMT and metastatic progression in HNSCC

To explore the role of HERC1 in Epithelial Mesenchymal Transition (EMT) in head and neck squamous cell carcinoma (HNSCC), we performed gene set enrichment analysis (GSEA) using TCGA-HNSC bulk RNA-seq data (Supplementary Fig. S3A). We performed Transwell migration and invasion assays. CD44⁺ cells derived from spheroids exhibited significantly greater migration and invasion capacities than the CD44^-^ spheroids (Supplementary Fig. S3B), suggesting an enhanced aggressiveness of the stem-like population. Moreover, CD44⁺ spheroids showed increased expression of EMT markers, including N-cadherin, and transcriptional regulators such as Snail and Slug, compared with CD44⁻ spheroids (Supplementary Fig. S3C), indicating a link between stemness and EMT.

**Fig. 3:**
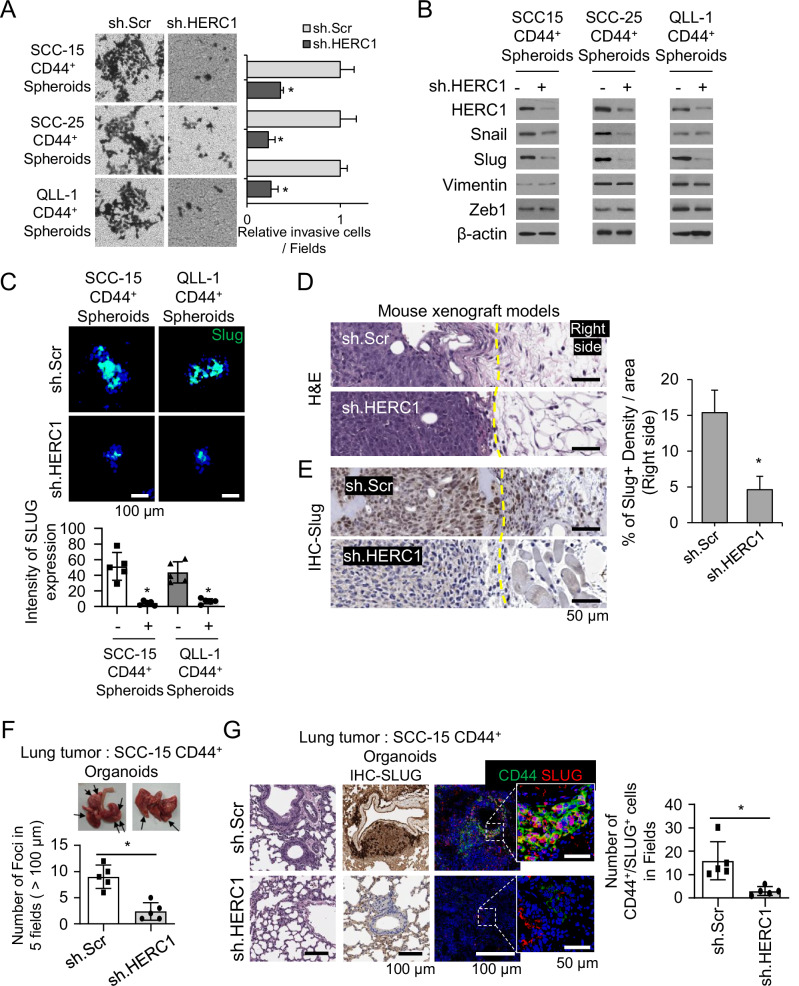
HERC1 promotes epithelial–mesenchymal transition (EMT) and metastasis in head and neck squamous cell carcinoma (HNSCC). **A** Representative images and quantification of Transwell invasion assays showing a marked reduction in invasive capacity of sh.HERC1-transduced CD44⁺ SCC15, SCC25, and QLL1 cells compared with control. **B** Western blot analysis of EMT-related proteins (Slug, Snail, Zeb1, and N-cadherin) in sh.Scr- and sh.HERC1-transduced CD44⁺ HNSCC cells. β-Actin serves as a loading control. **C** Immunocytochemistry staining of Slug (green) in CD44⁺ spheroids reveals decreased expression upon HERC1 knockdown. Nuclei were counterstained with DAPI (blue). Quantification of Slug intensity is shown on the right. Scale bars, 100 μm. **D** H&E staining of xenograft tumor sections shows that tumors derived from sh.HERC1 cells display well-defined, non-invasive borders, whereas control tumors (sh.Scr) exhibit irregular, locally invasive margins. **E** Graph and immunohistochemistry (IHC) staining for Slug in xenograft tumors reveals strong expression at the invasive front in control tumors, which is notably diminished in the sh.HERC1 group. Scale bars, 50 μm. **F** Representative gross lung tumor images (top) and quantification (bottom) showing fewer metastatic foci in mice injected with CD44⁺-derived organoids expressing sh.HERC1 compared to control. **G** H&E staining and immunofluorescence analysis of lung metastases reveal reduced expression of CD44 (green) and Slug (red) in sh.HERC1-injected mice relative to controls. Nuclei were counterstained with DAPI (blue). Magnified insets highlight metastatic lesions. Scale bars: main, 100 μm; insets, 50 μm.

HERC1 knockdown significantly reduced the migratory and invasive capacities of HNSCC CD44⁺ spheroids, as shown by Transwell assays (Fig. 3A). Western blot analysis revealed a consistent reduction in Slug expression across all HNSCC CD44⁺ spheroid models following HERC1 knockdown, while Snail expression was decreased in two of the cell models examined (Fig. 3B). Consistently, IF confirmed the loss of Slug expression in sh.HERC1 spheroids, whereas overexpression of HERC1 in CD44⁺ spheroids restored Slug levels (Fig. 3C), reinforcing the regulatory role of HERC1 in EMT.

In vivo, xenograft tumors generated from HERC1-silenced cells displayed well-circumscribed, noninvasive borders compared to the infiltrative pattern observed in control tumors (Fig. 3D). Immunohistochemistry (IHC) staining for Slug further supported this observation. Slug expression was prominently localized at the invasive margins of control tumors but was markedly diminished in sh.HERC1 tumors, indicating reduced EMT-driven invasion in the absence of HERC1 (Fig. 3E). Quantitative analysis confirmed an approximately 69.8% reduction in Slug-positive tumor cells following HERC1 knockdown, further supporting attenuated EMT-associated invasive behavior in vivo (Fig. 3E, Graph).

To assess the metastatic capacity in vivo, we used a tail vein injection model with CD44⁺ organoids [35]. Although control organoids formed multiple large metastatic foci in the lungs, HERC1 knockdown significantly reduced both the number and size of the metastatic lesions (Fig. 3F, G). Immunofluorescence confirmed that these metastatic lesions retained the expression of CD44 and Slug in control tumors but not in the HERC1-inhibited group (Fig. 3G). Collectively, these findings indicated that HERC1 promoted EMT and metastatic behavior in HNSCC by regulating CD44-associated stemness and EMT transcriptional programs.

### HERC1 inhibition reduces malignancy in co-culture with cancer organoids and CAFs

To mimic the Tumor Microenvironment (TME) and evaluate the interaction between cancer cells and stromal fibroblasts, we developed a co-culture system using HNSCC CD44⁺ organoids and primary fibroblasts. Normal fibroblasts were cultured in conditioned medium derived from HNC cells to induce a CAF-like phenotype. IF analysis confirmed the induction of CAF markers, FAP, SMA (α-smooth muscle actin), PDGFRα (Platelet-Derived Growth Factor Receptor α) in fibroblasts exposed to cancer-conditioned medium (Supplementary Fig. S4A).

**Fig. 4:**
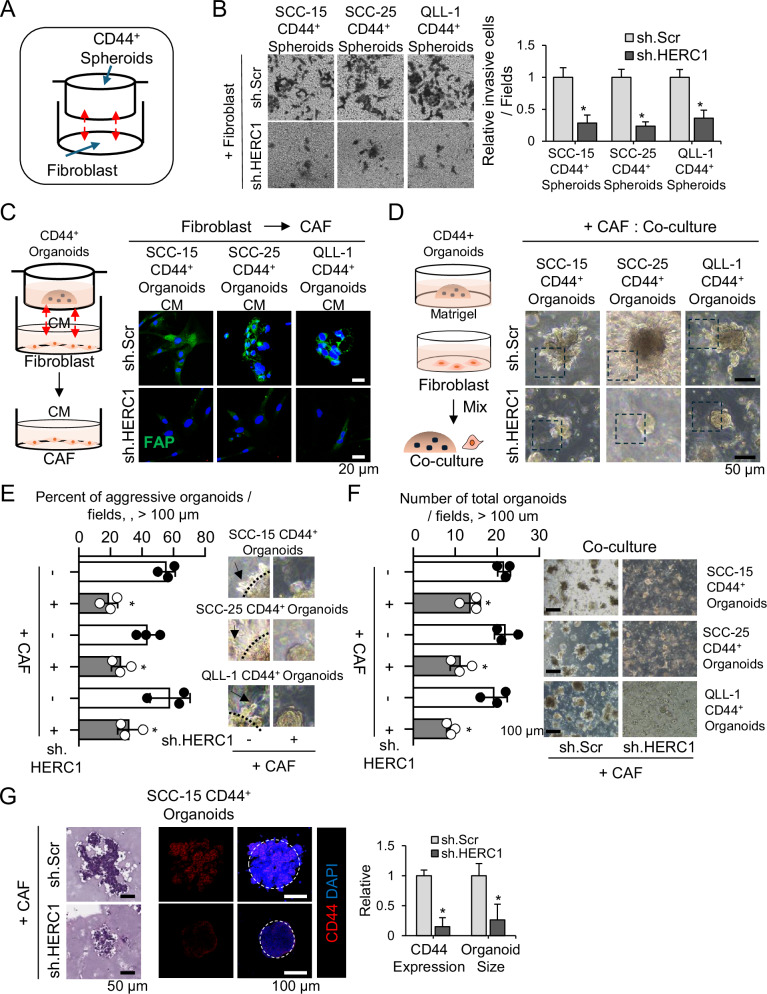
HERC1 inhibition suppresses fibroblast-induced malignancy in co-culture with CD44^+^ organoids. **A** Schematic representation of the indirect Transwell co-culture system, where CD44⁺ cells were seeded in the upper chamber and normal fibroblasts (WS1 cells) were seeded in the lower chamber. **B** Representative images and quantification of Transwell invasion assays showing that CAFs significantly enhance CD44⁺ spheroid invasion, which is markedly reduced upon HERC1 knockdown. **C** Indirect 3D co-culture system of fibroblasts and CD44⁺ organoids. Fibroblasts were cultured in the bottom chamber, while CD44⁺ organoids were embedded in Matrigel within the upper inserts. Immunofluorescence staining of fibroblast activation protein (FAP, green) confirming CAF-like activation of fibroblasts after co-culture with CD44⁺ cells. Nuclei were counterstained with DAPI (blue). Scale bars, 20 μm. **D** Representative bright-field images of CD44⁺ organoids co-cultured directly with fibroblasts in Matrigel. Aggressive, irregular, and protrusive organoid morphology was observed in controls but was absent in HERC1-knockdown organoids. Scale bars, 50 μm. **E** Magnified bright-field images highlighting protrusions and invasive edges in control organoids, which were absent in HERC1-deficient organoids. Scale bars, 50 μm. **F** Quantification of invasive organoid phenotypes under co-culture conditions with control or sh.HERC1 CD44⁺ organoids. Representative images of organoid invasion assays are shown on the right. Scale bars, 100 μm. **G** H&E and Immunofluorescence staining and quantification of CD44 expression in sh.HERC1-transduced organoids compared with controls. CD44 (red); nuclei were counterstained with DAPI (blue). Scale bars, 100 μm.

To assess the impact of CAFs on tumor invasion, we employed an indirect Transwell co-culture system in which fibroblasts were seeded in the lower chamber and CD44⁺ spheroids in the upper chamber (Fig. 4A). Co-culture with fibroblasts significantly enhanced the invasive capacity of CD44⁺ Spheroids; however, this pro-invasive effect was markedly suppressed when HERC1 was knocked down in cancer cells (Fig. 4B), indicating that HERC1 is required for CAF-mediated invasion.

We next established an indirect 3D co-culture model in which fibroblasts were placed in the bottom chamber and CD44⁺ organoids were embedded in Matrigel within the upper inserts. Under these conditions, control fibroblasts acquired CAF-like features with increased FAP expression, whereas HERC1-silenced fibroblasts failed to undergo this transition, indicating that HERC1 in HNC is associated with fibroblast-to-CAF conversion (Fig. 4C). Although activated fibroblasts are often described as having stellate or spindle-like morphology, their appearance can vary depending on culture conditions, extracellular matrix context, and paracrine signaling. The fibroblasts were cultured under such conditions, which likely contributed to the observed morphological variability (Fig. 4C). Importantly, activation status was evaluated based on functional behavior within the model rather than morphology alone; therefore, despite lacking a textbook stellate shape, the cells shown in Fig. 4C exhibit functional characteristics consistent with activated CAFs.

In contrast, HERC1 knockdown substantially suppressed these CAF-associated morphological and invasive changes, and the organoids retained more compact, rounded structures (Fig. 4D–F). Histological analysis and IF staining further supported these findings, with aggressive invasive fronts observed in the control co-culture condition that were notably diminished in the HERC1-silenced group (Fig. 4E, G).

Collectively, these results demonstrate that HERC1 plays a pivotal role in promoting tumor–stromal crosstalk and enhancing the invasive phenotype of CD44⁺ organoids in response to CAF-derived signals. Targeting HERC1 may disrupt this interaction and mitigate the pro-metastatic influence of the TME.

### IL-6 regulates stemness and tumorigenesis in CD44^+^ spheroids and cancer organoids of head and neck cancer

To investigate how the tumor microenvironment (TME) contributes to the maintenance of cancer stem-like cells (CSCs), we sought to identify key paracrine factors that promote stemness in head and neck cancer[10, 36]. Among candidate cytokines known to be enriched in the TME, Interleukin-6 (IL-6) emerged as a promising target due to its established role in inflammation-driven tumor progression and stem cell maintenance[37].

**Fig. 5:**
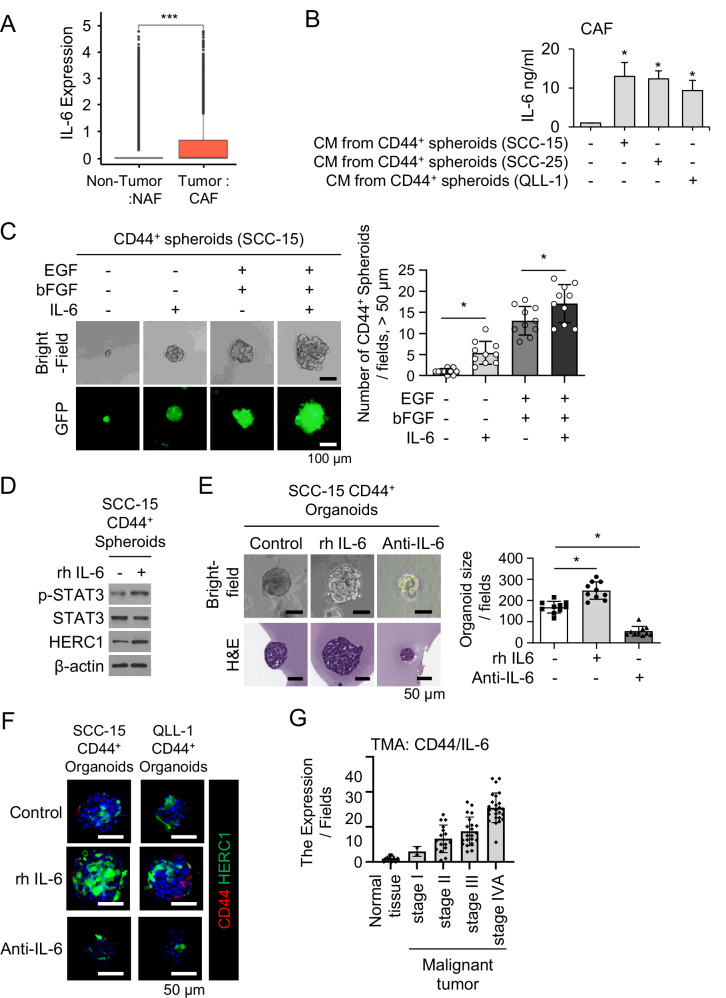
Interleukin-6 (IL-6) regulates stemness and tumorigenesis in CD44^+^ cells and cancer organoids via the JAK/STAT3/HERC1 axis. **A** Box plot showing significantly higher IL-6 expression in tumor cells compared to normal cells in HNSCC samples from single-cell RNA-seq dataset. **B** Enzyme-linked immunosorbent assay (ELISA) confirming increased IL-6 concentrations in conditioned media from fibroblast–cancer cell co-cultures compared to controls. **C** Representative bright-field and fluorescence images of CD44⁺ spheroids treated with increasing concentrations of recombinant IL-6, showing enhanced spheroid size and growth in a dose-dependent manner. Scale bars, 100 μm. **D** Western blot analysis of p-STAT3 and HERC1 expression in CD44⁺ spheroids and organoids following IL-6 stimulation. **E** Quantification of SCC-15 CD44⁺ organoids diameters under IL-6 treatment conditions. Data represent mean ± SD (*n* = 3). Bright-field and hematoxylin and eosin (H&E) staining images of organoids treated with control IgG, recombinant IL-6, or IL-6-neutralizing antibody. Scale bars, 50 μm. **F** Immunofluorescence showing co-localization of HERC1 (green) and CD44 (red) in control IgG, recombinant IL-6, or IL-6-neutralizing antibody-treated organoids. Scale bars, 50 μm. **G** CD44 and IL-6 expression levels assessed by immunofluorescence and tissue microarray (TMA) analysis.

To evaluate whether IL-6 acts upstream of the HERC1/CD44 axis, we extended our analysis beyond fibroblast subtype comparison. Single-cell RNA-seq profiling revealed that IL-6 expression was markedly elevated in cancer-associated fibroblasts (CAFs) compared with normal-associated fibroblasts (NAFs) (Fig. 5A). To assess whether IL-6 expression is associated with stemness, we analyzed single-cell RNA-seq data based on patient-level fibroblast IL-6 expression and found a positive correlation between IL-6^High^ and CD44 expression, supporting a transcriptional association between IL-6 signaling and stem-like features in tumors (Supplementary Fig. S5A).

In vitro, IL-6 emerged as a prominent cytokine secreted by CAFs, with elevated expression observed in fibroblasts exposed to cancer-conditioned medium (Supplementary Fig. S5B).

Enzyme-linked immunosorbent assay (ELISA) demonstrated that conditioned medium derived from CD44⁺ cells effectively activated CAFs, resulting in a marked increase in IL-6 secretion [36], whereas conditioned medium from CD44⁻ cells failed to elicit a comparable response, indicating that IL-6 induction in CAFs is specifically driven by CD44⁺ cell–CAF crosstalk (Fig. 5B, Supplementary Fig. S5C). Consistently, western blot analysis confirmed a strong upregulation of IL-6 protein in CAFs treated with CD44⁺ cell–derived conditioned medium, further supporting the selective activation of IL-6 signaling by CD44⁺ tumor cells (Supplementary Fig. S5D).

To determine the functional relevance of IL-6 in modulating stemness, we treated CD44⁺ spheroids with recombinant IL-6 and observed a dose-dependent increase in spheroid size, consistent with enhanced self-renewal and stemness (Fig. 5C). Quantitative analysis showed a significant increase in spheroid diameter in the IL-6-treated groups compared to the controls (Fig. 5C).

Mechanistically, IL-6 treatment activated the JAK/STAT3 pathway and upregulated HERC1 expression in both the spheroids and organoids, as confirmed by western blotting analysis (Fig. 5D). Conversely, treatment with IL-6 neutralizing antibody inhibited organoid growth and suppressed stemness (Supplementary Fig. S5E, Fig. 5E).

Immunofluorescence analysis showed that recombinant IL-6 markedly increased the expression of both HERC1 and CD44 in CD44⁺ organoids, whereas IL-6 neutralizing antibody treatment substantially reduced their expression (Fig. 5F).

Consistent with these organoid findings, analysis of head and neck cancer tissue microarrays (TMAs) revealed that IL-6 and CD44 expression levels were significantly elevated in high-grade tumors compared with low-grade counterparts, with a positive correlation between IL-6 intensity and CD44 expression (Fig. 5H). These findings collectively demonstrate that CAF-derived IL-6 promotes stemness and tumorigenic potential in CD44⁺ cells and CD44^+^-derived organoids via the IL-6/p-STAT3/HERC1 signaling axis, thus establishing a mechanistic link between inflammatory signaling and cancer stemness in the head and neck TME.

### HERC1 regulates a STAT3-dependent p-ERK signaling axis in CD44^+^ HNSCC cells

To investigate the mechanistic relationship between CD44 and HERC1, we performed additional validation experiments. Previous studies have shown that ERK signaling regulates CD44 and contributes to cancer stemness and EMT [38, 39], prompting us to examine MAPK pathway involvement downstream of HERC1[27, 40]. Following HERC1 inhibition in CD44⁺ cells, Western blot analysis revealed reduced phosphorylation of ERK, whereas total ERK levels remained unchanged (Supplementary Fig. S6A). In contrast, p-JNK and p-p38 levels were not significantly affected, suggesting pathway specificity. To determine whether ERK acts upstream of HERC1, we treated cells with the ERK inhibitor U0126; however, ERK inhibition did not alter HERC1 expression, indicating that HERC1 functions upstream of ERK rather than being regulated by it (Supplementary Fig. S6B). Next, pharmacological inhibition of STAT3 using WP1066 markedly reduced phosphorylated STAT3 levels and resulted in a parallel decrease in HERC1 expression, indicating that HERC1 upregulation is STAT3-dependent (Supplementary Fig. S6C). Immunofluorescence analysis further confirmed this regulatory axis, showing strong co-expression and spatial colocalization of HERC1 and CD44 in organoids, whereas WP1066 treatment led to diminished HERC1 signal and reduced CD44 expression (Supplementary Fig. S6C). Together, these findings define a functional STAT3–HERC1–p-ERK signaling axis and position HERC1 as a mechanistic mediator linking STAT3 signaling to ERK-dependent stemness in CD44⁺ HNSCC cells.

### IL-6/STAT3/HERC1 signaling sustains drug resistance to cisplatin and 5-FU in Co-culture systems

**Fig. 6:**
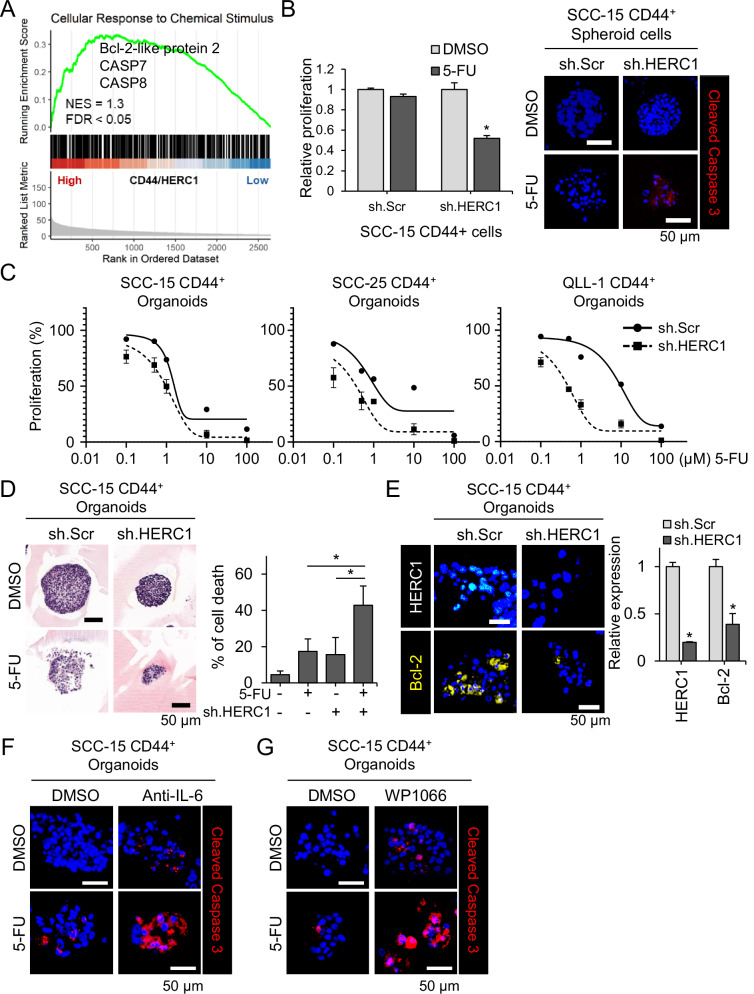
Interleukin-6 (IL-6)/STAT3 signaling sustains drug resistance to 5-fluorouracil (5-FU) in CD44^+^ organoids. **A** Gene set enrichment analysis (GSEA) showing enrichment of apoptosis-related genes (BCLL2, CASP7, CASP8) in high CD44/HERC1 tumors. **B** Cell viability and apoptotic cell death in SCC15 CD44⁺ Spheroid cells following treatment with 5-FU. Cell viability was measured by WST-8 assay, and apoptosis was assessed by immunofluorescence staining for cleaved caspase-3 (red), with nuclei counterstained using DAPI (blue). Representative images of treated spheroids are shown on the right. Scale bars, 50 μm. **C** Dose–response curves of CD44⁺ organoids treated with increasing concentrations of 5-fluorouracil (5-FU) for 72 h in all HNSCC cells. HERC1 knockdown (sh.HERC1) significantly reduced organoid viability compared to control (sh.Scr) in a dose-dependent manner. **D** Representative hematoxylin and eosin (H&E) stained images of organoids treated with DMSO and 5-FU. Scale bars, 50 μm. **E** Immunofluorescence staining of HERC1 and Bcl-2 (anti-apoptotic marker) in SCC-15 CD44^+^ organoids. Quantification graphs for Bcl-2 and HERC1 intensity are shown on the right. Scale bars, 50 μm. **F** Immunofluorescence staining of cleaved Caspase-3 (red) and DAPI (blue) in SCC-15 CD44^+^ organoids treated with 5-FU in the presence of either DMSO or anti-IL-6 antibody, or **G** in combination with a STAT3 inhibitor. Scale bars, 50 μm. *p* < 0.05.

To determine whether HERC1 contributes to drug resistance in cancer stem-like cells, we evaluated the chemotherapeutic response of CD44⁺ organoids treated with 5-FU and cisplatin in co-culture settings. First, gene set enrichment analysis of TCGA HNSCC datasets revealed that high CD44/HERC1 expression was positively associated with the apoptosis related gene signature, suggesting a potential role for anti-apoptotic signaling in therapy resistance (Fig. 6A).

Consistent with this, drug sensitivity assays demonstrated that CD44⁺ cells exhibited reduced responsiveness to standard chemotherapeutic agents compared with CD44⁻ cells, with significantly higher survival rates following cisplatin and 5-fluorouracil (5-FU) treatment (Supplementary Fig. S7A). HERC1-silenced CD44⁺ cells (sh.HERC1) exhibited significantly greater sensitivity to both agents compared to CD44⁻ cells (Supplementary Fig. S7B, Fig. 6B).

In 2D cell cultures, sh.HERC1 cells showed only a modest increase in cisplatin sensitivity compared with control cells (Data not shown). In contrast, CD44⁺-derived organoids, which better recapitulate clinical conditions, exhibited a markedly greater reduction in viability upon HERC1 depletion, indicating enhanced cisplatin responsiveness (Fig. 6C). Bright-field and H&E staining of organoid sections revealed decreased size and structural integrity following treatment, especially in the HERC1-deficient groups (Fig. 6D).

To examine whether HERC1 modulated apoptosis in response to chemotherapy, we performed immunofluorescence staining for cleaved Caspase-3 (a pro-apoptotic marker) and Bcl-2 (an anti-apoptotic marker). Organoids with HERC1 knockdown showed increased cleaved Caspase-3 and reduced Bcl-2 expression compared to the controls, confirming enhanced apoptotic activity (Fig. 6E). Next, we investigated whether IL-6 signaling contributed to this resistance. Co-treatment with 5-FU and either an anti-IL-6 neutralizing antibody or a STAT3 inhibitor resulted in a synergistic increase in cell death, as evidenced by elevated cleaved Caspase-3 levels in the organoids (Fig. 6F, G). These results support a model in which IL-6–mediated STAT3 activation induces HERC1 expression, thereby promoting chemoresistance in CD44⁺ cancer organoids. Targeting the IL-6/STAT3/HERC1 axis could overcome resistance in cancer stem-like cells and improve therapeutic efficacy in head and neck cancer.

### Effect of HERC1 inhibition on mouse xenograft models and human tumor samples

**Fig. 7:**
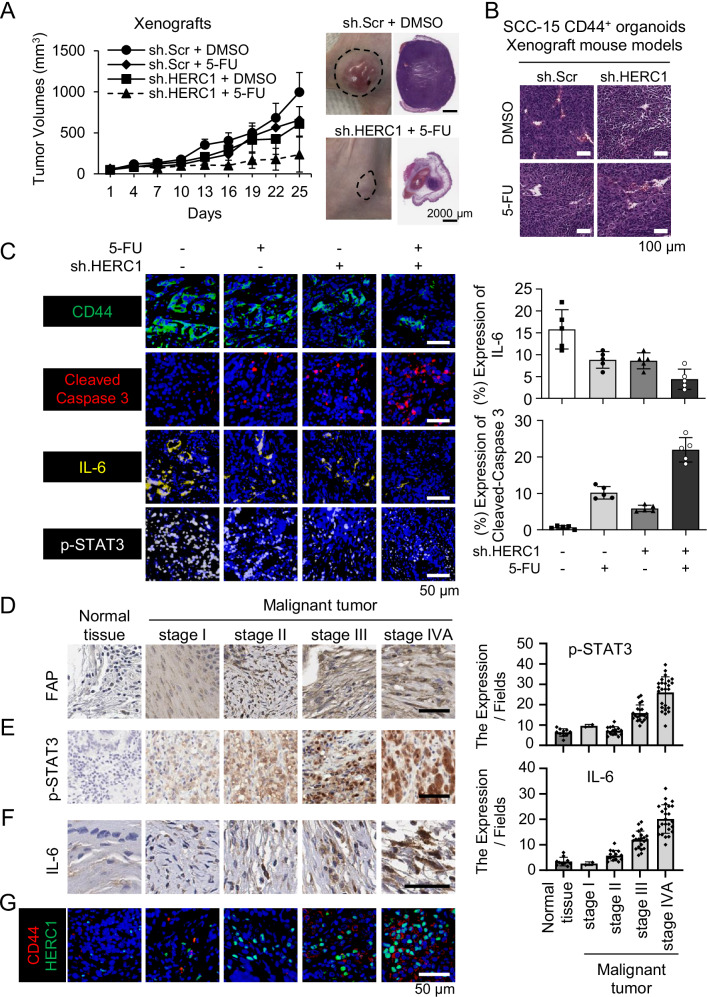
HERC1 inhibition suppresses tumor growth in xenograft models and correlates with malignant features in human head and neck cancer tissues. **A** Tumor volume analysis in mouse xenograft models. SCC-15 CD44^+^ organoids (5 × 10⁶) were subcutaneously injected into nude mice and treated with control, sh.HERC1, 5-fluorouracil (5-FU), or combination therapy. Representative gross tumor images and corresponding H&E-stained sections are shown. Scale bars: 2 mm (gross), 50 μm (H&E). **B** Higher-magnification H&E images of tumor tissues from (**A**), showing histological differences between treatment groups. Scale bars, 100 μm. **C** Representative immunofluorescence staining of mouse tumor sections for CD44 (green), cleaved Caspase-3 (red), IL-6 (yellow), and phosphorylated STAT3 (p-STAT3, white). DAPI (blue) was used for nuclear staining. Scale bars, 50 μm. **D**–**G** Immunohistochemical analysis of human HNSCC tissue microarray (TMA) samples across different tumor grades, stained for HERC1, IL-6, p-STAT3, CD44, and fibroblast activation protein (FAP). Scale bars, 50 μm.

To evaluate the therapeutic effects of HERC1 inhibition in vivo, we established mouse xenograft models using HNSCC CD44^+^ organoids, with or without HERC1 knockdown. The mice were divided into the following treatment groups: control, HERC1 knockdown (sh.HERC1), 5-FU, and combination therapy (sh.HERC1 and 5-FU). Tumor volumes in the sh.HERC1 group were reduced by approximately 38.8% compared to the controls, whereas 5-FU treatment alone reduced the tumor size by 34.1%. Notably, combination treatment with HERC1 inhibitor and 5-FU resulted in a synergistic effect, reducing tumor volume by more than 76.4% (Fig. 7A). Histological analysis of tumor tissues by H&E and IF staining confirmed enhanced apoptosis and reduced CD44 expression in tumors from the combination group (Fig. 7A–C). IF showed increased cleaved Caspase-3 and decreased CD44 and IL-6 expression in sh.HERC1 and combination-treated tumors, supporting the induction of cell death and loss of cancer stemness under HERC1-targeted therapy (Supplementary Fig. S8A, Fig. 7C).

To evaluate the clinical relevance of HERC1 in head and neck cancer, we examined the expression of FAP, p-STAT3, IL-6, HERC1, and CD44 using a commercially purchased human head and neck tumor microarray (TMA) that included samples representing multiple tumor grades. IHC analysis revealed that p-STAT3, IL-6, and HERC1 levels were markedly elevated in high-grade tumors compared with low-grade counterparts, with quantification confirming a positive correlation between their expression and tumor stage, and the representative images correspond to stromal regions within tumor-containing TMA cores (Supplementary Fig. S8B, Fig. 7D–F). Immunofluorescence further demonstrated strong co-localization of HERC1 and CD44 in advanced tumors (Fig. 7G), supporting the link between IL-6/p-STAT3/HERC1 signaling and CSC marker expression in aggressive disease.

Collectively, these results demonstrated that HERC1 inhibition not only suppressed tumor growth in vivo but also correlated with key markers of stemness and tumor aggressiveness in human tissues. These findings support the potential of HERC1 as a clinically relevant therapeutic target for head and neck cancers.

## Discussion

This study has identified HERC1 as a previously unrecognized and critical regulator of cancer stem-like properties in HNSCC, particularly within CD44⁺-enriched spheroid and organoid models. Elevated HERC1 expression in CD44⁺ HNSCC cells was associated with stem-like phenotypes. Functional studies demonstrated that HERC1 knockdown significantly impaired spheroid growth, organoid formation, EMT, and in vivo tumorigenicity, establishing its role in maintaining tumor-initiating capacity (Supplementary Fig. S8C).

Although our findings strongly support a functional relationship between STAT3 signaling and HERC1 upregulation, the precise transcriptional mechanism remains to be experimentally validated. In this study, we demonstrated that IL-6 stimulation and CAF co-culture activate STAT3 and subsequently increase HERC1 expression, establishing a regulatory connection within the IL-6/STAT3/HERC1 axis. However, whether STAT3 directly binds to the HERC1 promoter or regulates HERC1 transcription through intermediate transcriptional co-factors has not yet been determined. Techniques such as ChIP-qPCR or ChIP-seq would be required to determine direct STAT3-DNA interactions and identify putative STAT3-responsive elements within the HERC1 promoter region. Future mechanistic studies employing these approaches will be essential to clarify whether HERC1 is a direct transcriptional target of STAT3 or regulated through indirect signaling cascades. Despite this limitation, our results provide a compelling framework linking inflammatory signaling to HERC1-driven cancer stemness and invasive behavior, laying the groundwork for deeper transcriptional interrogation in subsequent studies.

Mechanistically, HERC1 expression was induced by IL-6 secreted by CAFs via the JAK/STAT3 pathway. This positions HERC1 as a key downstream effector of microenvironmental signaling and highlights a functional axis, IL-6/STAT3/HERC1, that sustains cancer stemness and contributes to chemoresistance. Notably, HERC1 knockdown not only suppressed CSC traits but also sensitized CD44⁺ organoids to 5-FU, triggering apoptosis through cleaved Caspase-3 activation and Bcl-2 downregulation. These findings underscore the dual role of HERC1 as an intrinsic modulator of CSC identity and a mediator of extrinsic TME-derived cues.

The TME plays a central role in HNSCC progression, influencing plasticity and resistance through paracrine factors such as IL-6 [14, 15]. Using HNSCC cell lines (SCC-15, SCC-25, and QLL-1), we demonstrated that HERC1 depletion consistently reduced spheroid and organoid formation. This loss of self-renewal capacity was accompanied by the downregulation of core pluripotency genes, including Nanog, Sox-2, Oct4, and c-Myc, further confirming HERC1’s role in maintaining stemness.

In support of this mechanism, both CAF co-culture and IL-6 treatment significantly upregulated HERC1 expression in CD44⁺ cells. These data revealed that inflammatory cytokines within the TME reprogrammed cancer cells by modulating HERC1 levels, thereby linking stromal signals to CSC maintenance. We propose that HERC1 serves as a molecular integrator of the extrinsic IL-6/STAT3 signaling and intrinsic CSC programs, promoting both cellular plasticity and therapeutic resistance.

The clinical relevance of this axis is supported by our tissue microarray data, which showed that HERC1 and CD44 expression were co-upregulated in higher-stage HNSCC specimens. This correlation suggests that the HERC1–CD44 pathway may represent a conserved feature of aggressive disease and a potential prognostic biomarker. Moreover, the enhanced sensitivity to 5-FU following HERC1 depletion suggests a therapeutic opportunity to target CSCs and overcome drug resistance, which is an enduring challenge in the treatment of HNSCC.

Nonetheless, our study has some limitations. Although we used patient-derived organoids and in vivo xenograft models, the long-term effects of HERC1 inhibition on metastasis and recurrence remain unknown. Additional studies are warranted to delineate the upstream transcriptional regulation of HERC1 by STAT3 and to explore potential compensatory pathways.

In summary, our findings establish HERC1 as a key mediator of cancer stem cell plasticity, microenvironmental responsiveness, and chemoresistance in HNSCC. By uncovering a novel IL-6/STAT3/HERC1 signaling axis that sustains CD44⁺ cell maintenance, this study provides a conceptual framework for disrupting CSC-supportive niches. Targeting HERC1 may represent a promising strategy for attenuating stemness, impairing tumor growth, and enhancing the efficacy of conventional therapies for aggressive HNSCC.

This study demonstrates that HERC1 was a critical mediator of cancer stemness and chemoresistance in HNSCC. IL-6 secreted by CAFs activated STAT3 signaling, which upregulated HERC1 and enhanced CD44⁺ organoid growth, EMT, and drug resistance. HERC1 knockdown reduced stemness markers, impaired tumor formation and metastasis, and significantly sensitized tumors to 5-FU and cisplatin. Clinical samples showed an increased expression of HERC1, CD44, and FAP in patients with advanced HNSCC. Targeting the IL-6/STAT3/HERC1 axis may be an effective strategy for eliminating cancer stem cells and overcoming chemoresistance in HNSCC.

## Supplementary information

Supplementary Material

## Data Availability

The data supporting the findings of this study are available from the corresponding author upon reasonable request.
