# Supplementary material for: HERC1 oncogene enhances stemness and tumorigenic potential in CD44+-derived organoids of head and neck squamous cell carcinoma through IL-6/STAT3 signaling
Source: Oncogene. 2026 Apr 11;45(19):1840–55. doi: 10.1038/s41388-026-03725-9 (PMC13139034; doi:10.1038/s41388-026-03725-9)

Supplementary Figure Legends

Supplementary Figure S1. Tissue microarray (TMA) HN802d containing 80 human head and neck tissue cores, including normal and malignant specimens from various anatomical sites, was used for histological evaluation. (A) Kaplan–Meier survival curves stratified by CD44 and HERC1 co-expression levels. Patients with high $CD44^{High}/HERC1^{High}$ expression show a trend toward poorer overall survival (TNM I-II; $p = 0.107$, TNM IV; $p = 3.4e-06$). (B) TMA map indicating tissue origin and pathological classification of each core. Malignant samples include laryngeal (Lar), pharyngeal (Pha), nasal (Nos), maxillary (Max), piriform (Pir), glottic (Glo), oral (Che), hypopharyngeal (Hyp), tongue (Ton), parotid (Par), and facial (Fac) carcinomas. Normal tissues (green) are derived from larynx (Lar), epiglottis (Epi), tonsil (Ton), submandibular gland (Sub), and other adjacent structures. (C) Representative hematoxylin and eosin (H&E) stained image of the TMA slide, showing the histopathological features of each tissue core. This platform enables high-throughput analysis of tumor heterogeneity, histological subtype, and tumor microenvironment in head and neck cancers.

Supplementary Figure S2. (A) Gene set enrichment analysis (GSEA) showing enrichment of cancer stemness-related genes in the high CD44/HERC1-expressing group of HNSCC samples. (B) Western blot analysis of HERC1 knockdown efficiency in SCC-15, SCC-25, and QLL-1 cells using anti-HERC1 antibodies. β-Actin was used as a loading control. (C) Cell growth curves of control and HERC1 knockdown cells were assessed over 72 hrs. using a cell viability assay. Data are presented as mean ± SD ($n = 3$). (D) Representative bright-field images of monolayer cultures (Parent cells; top) and spheroids (Cancer stem-like cells; bottom) formed under spheroid-inducing conditions in SCC-15, SCC25, and QLL-1 cells. Scale bars, 50 μm. (E) Western blot analysis of stemness markers (SOX-2, OCT4, NANOG, and c-Myc) and the CSC marker (CD44) in $CD44^{-}$ cells and $CD44^{+}$ cells derived from spheroid cells. (F) representative bright-field images of $CD44^{+}$-derived organoids with or without HERC1

knockdown. Scale bars, 100 μm. (G) Immunofluorescence staining showing CD44 (green) expression in $CD44^+$-derived organoids following HERC1 knockdown (sh.HERC1). Nuclei were counterstained with DAPI (blue). Scale bars, 50 μm. (H) Western blot analysis shows reduced stemness markers (SOX2, OCT4, NANOG, and CD44) following HERC1 knockdown. (I) Representative bright-field images and quantification demonstrate impaired spheroid formation in HERC1-silenced $CD44^+$ cells. Scale bar: 50 μm.

Supplementary Figure S3. (A) Gene set enrichment analysis (GSEA) showing significant enrichment of EMT-related genes in the high CD44/HERC1-expressing group compared to the low CD44/HERC1 group in HNSCC samples. (B) Transwell invasion assay comparing the invasive capacity of $CD44^+$ and $CD44^-$ HNSCC cells. Representative bright-field images (top) and quantification of invaded cells (bottom) are shown. (C) Western blot analysis of epithelial–mesenchymal transition (EMT) markers (E-cadherin, N-cadherin, Vimentin, Snail, and Slug) in $CD44^+$ and $CD44^-$ cell populations from SCC-15, SCC25, and QLL-1 cell lines. β-Actin was used as a loading control.

Supplementary Figure S4. Immunofluorescence staining of fibroblast activation protein (FAP), SMA (Smooth Muscle Actin), and Platelet-Derived Growth Factor α (PDGFα) in the fibroblast cell line WST1 cultured under SCC-15-conditioned media. Nuclei are counterstained with DAPI (blue). Scale bars: 20 μm.

Supplementary Figure S5. (A) Boxplot analysis of TCGA-HNSC dataset shows significantly higher IL-6 expression in CD44-high tumors compared with CD44-low tumors ($p < 0.001$). (B) Schematic of the indirect co-culture workflow. Conditioned medium from cancer spheroid cultures was collected and transferred to fibroblasts. After 24 h and 48 h of exposure, fibroblast-

conditioned medium (CM) and cell pellets were harvested for subsequent assays, including western blotting and ELISA. (C) Enzyme-linked immunosorbent assay (ELISA) confirming increased IL-6 concentrations in conditioned media from fibroblast–$CD44^-$ cell co-cultures compared to controls. (D) Western blot analysis of IL-6 protein levels in Cancer associated with fibroblast. β-actin was used as a loading control. (E) Representative bright-field and hematoxylin and eosin (H&E) staining images of established $CD44^+$-derived organoids. Scale bars: 50 μm.

Supplementary Figure S6. (A) Western blot analysis demonstrating reduced p-ERK1/2 levels following HERC1 knockdown in $CD44^+$ cells, while total ERK remains unchanged. (B) Treatment with the ERK inhibitor U0126 did not alter HERC1 expression, indicating that HERC1 functions upstream of ERK activation. (C) Western blot and immunofluorescence analyses show that treatment with the STAT3 inhibitor WP1066 reduces p-STAT3, HERC1, and CD44 levels in $CD44^+$ organoids. Scale bar: 50 μm.

Supplementary Figure S7. (A) Cell viability and apoptotic cell death in SCC15 and SCC25 $CD44^-$ and $CD44^+$ spheroids following treatment with 5-FU. Cell viability was measured by WST-8 assay, and apoptosis was assessed by immunofluorescence staining for cleaved caspase-3 (red), with nuclei counterstained using DAPI (blue). Representative images of treated spheroids are shown on the right. Scale bars, 20 μm. (B) Cell proliferation of SCC-25 $CD44^+$ cells following treatment with sh.HERC1 or 5-FU, assessed by WST-8 assay. Representative bright-field images of treated $CD44^+$ cells are shown on the right. Scale bars, 20 μm.

Supplementary Figure S8. (A) Immunofluorescence analysis of CD44 and phosphorylated STAT3 (p-STAT3) expression in xenograft tumor tissues following treatment with sh.HERC1 and/or 5-fluorouracil (5-FU). (B) Quantification of fibroblast activation protein (FAP) expression in cancer-associated fibroblasts (CAFs) using tissue microarray (TMA) analysis. (C) Schematic model illustrating the proposed mechanism by which CAF-derived IL-6 promotes cancer progression through activation of the IL-6–STAT3–HERC1–ERK signaling axis in $CD44^+$ cancer stem-like cells and $CD44^+$ organoids. (1) In the tumor microenvironment, IL-6 secreted from CAFs activates STAT3 phosphorylation, which in turn upregulates HERC1 expression in $CD44^+$ cells. Elevated HERC1 subsequently drives ERK activation, reinforcing stemness, self-renewal, and survival signaling. (2) Upon differentiation *in vitro*, $CD44^+$-derived organoids embedded in Matrigel retain the molecular characteristics of primary $CD44^+$ tumor cells and faithfully model IL-6–responsive pathways. Activation of the STAT3–HERC1–ERK axis promotes proliferation, epithelial–mesenchymal transition (EMT), and therapeutic resistance. This CAF–CSC crosstalk highlights the IL-6–STAT3–HERC1–ERK pathway as a mechanistically defined therapeutic vulnerability in HNSCC.

Supplementary Figure S1

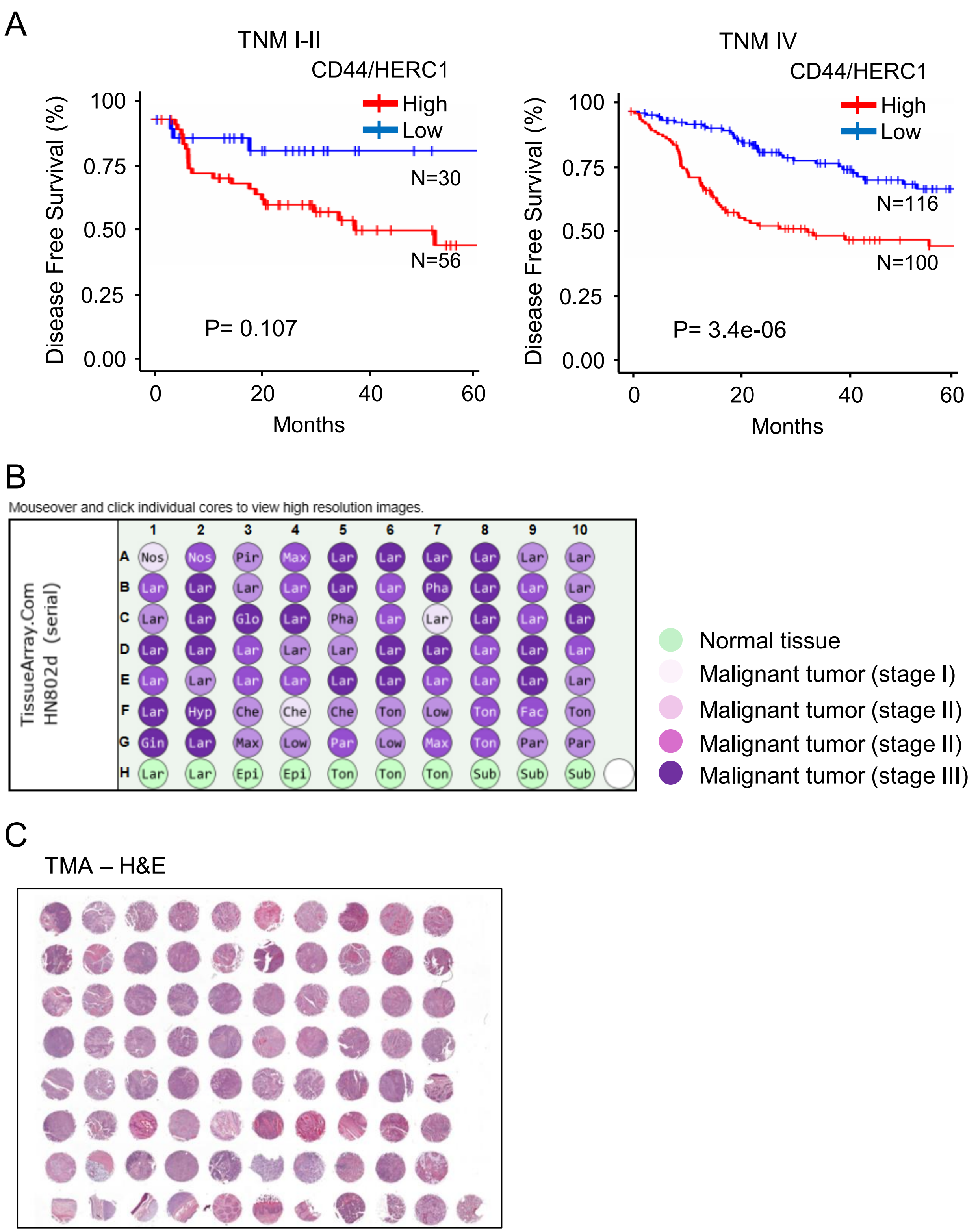

Supplementary Figure S2

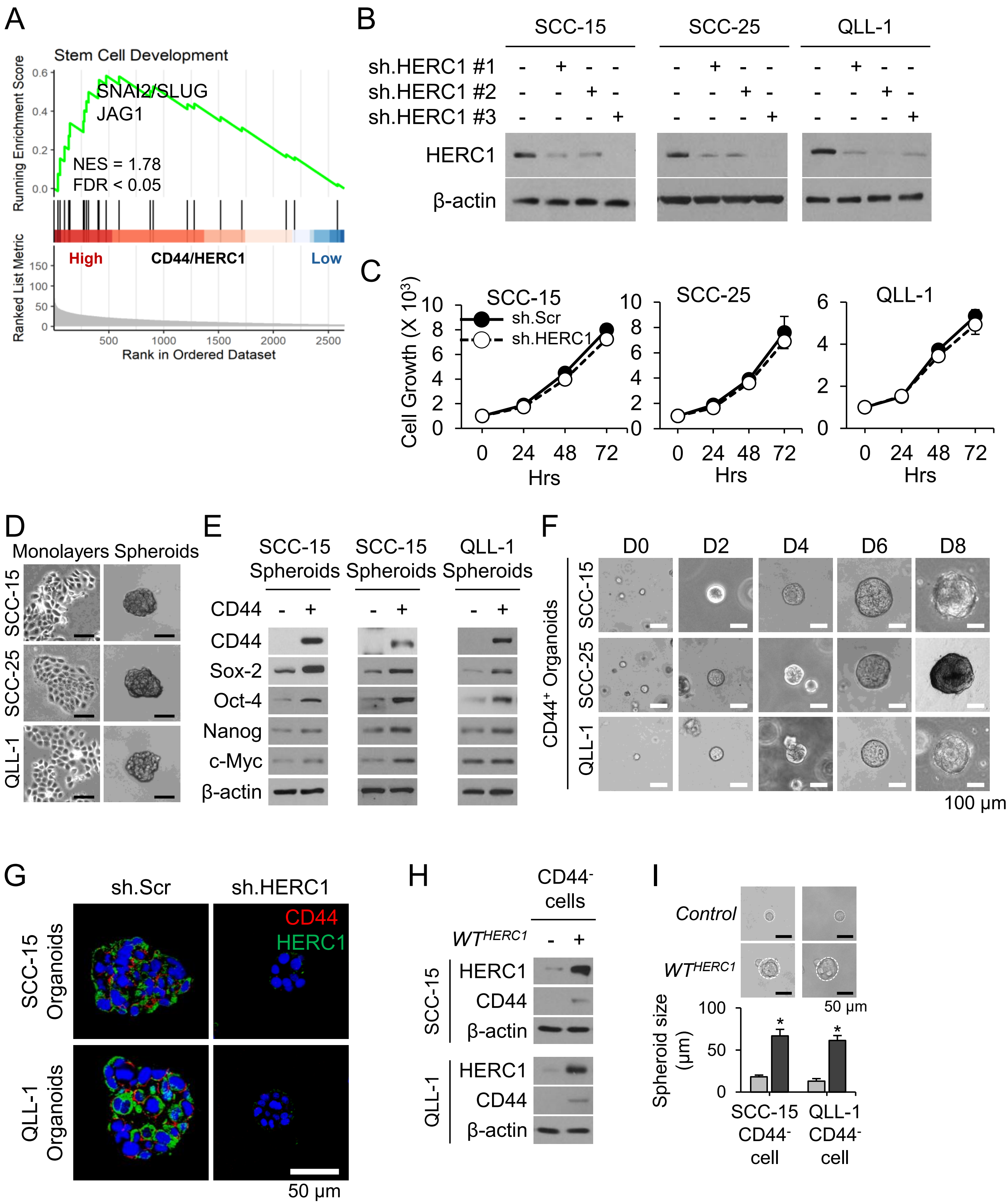

Supplementary Figure S3

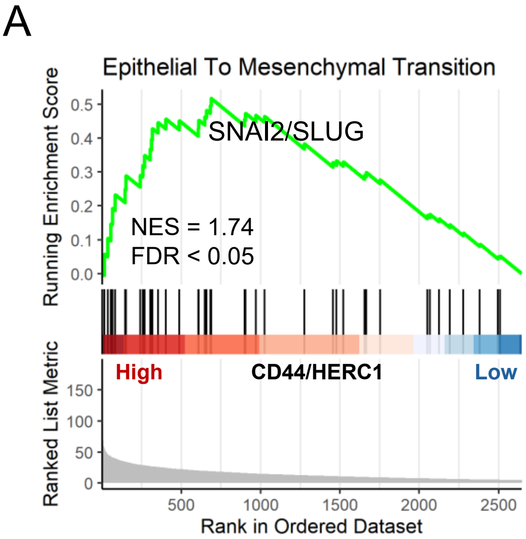

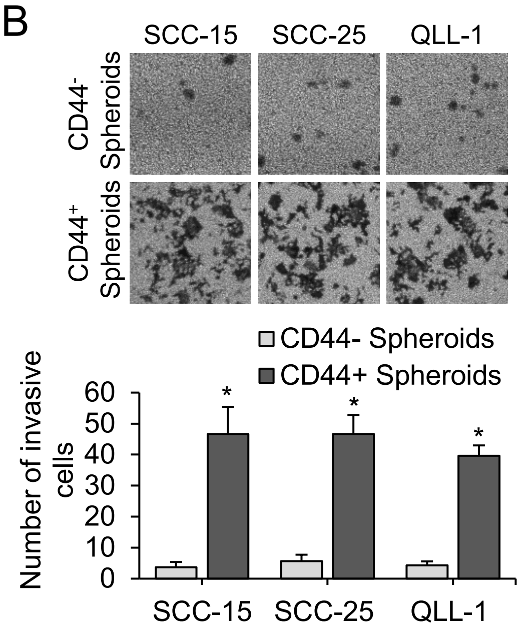

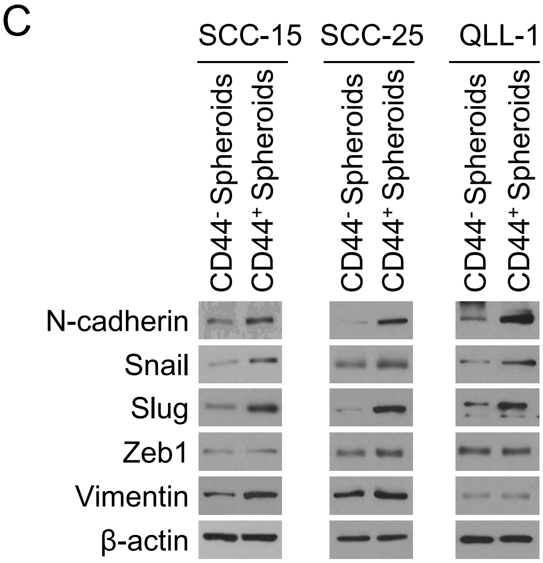

Supplementary Figure S4

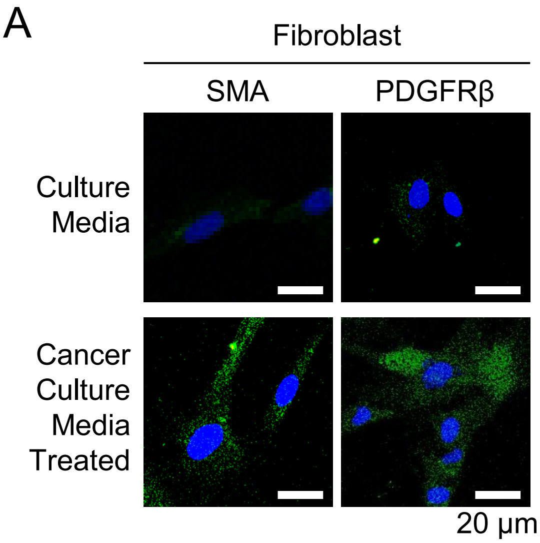

Supplementary Figure S5

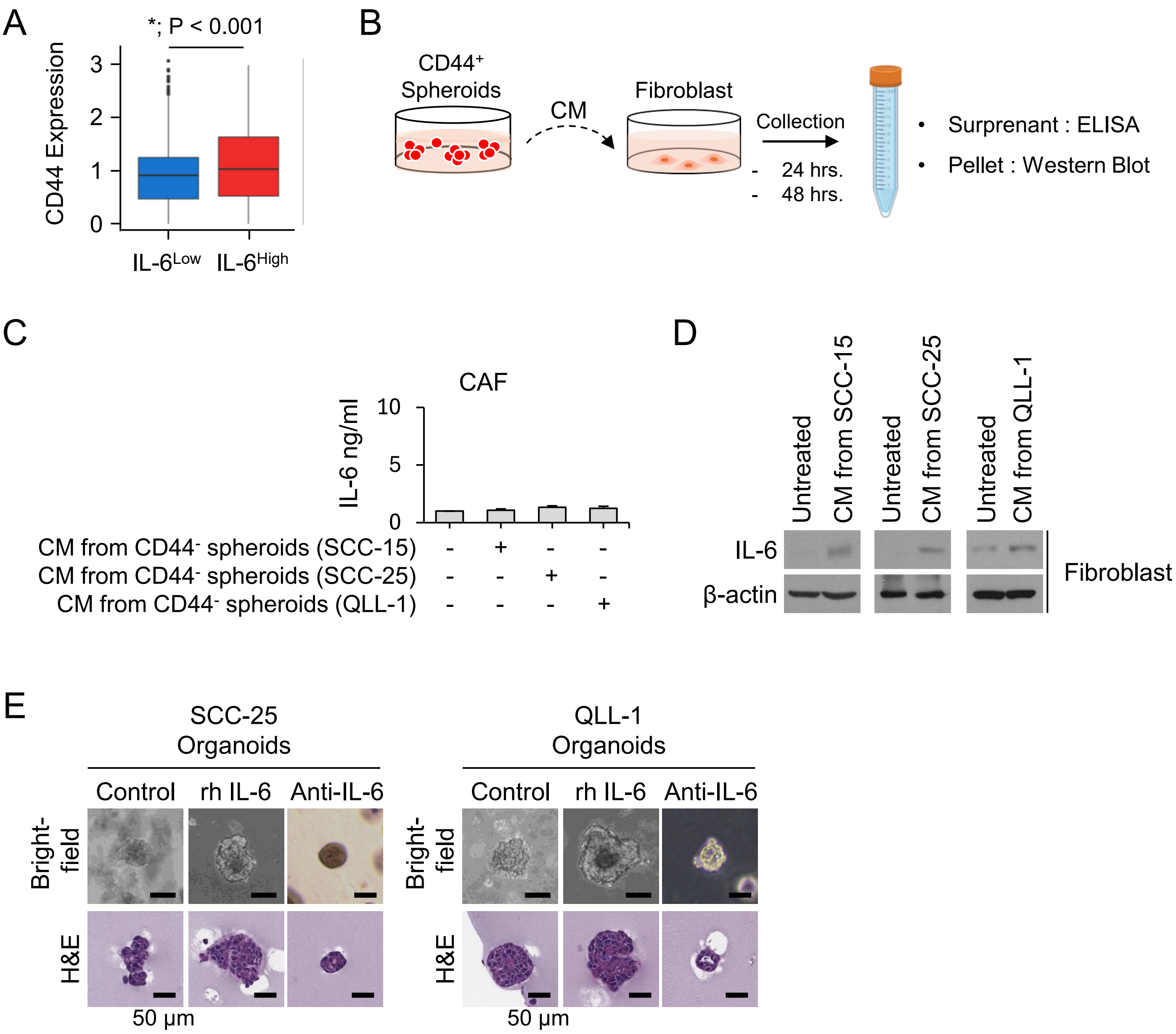

Supplementary Figure S6

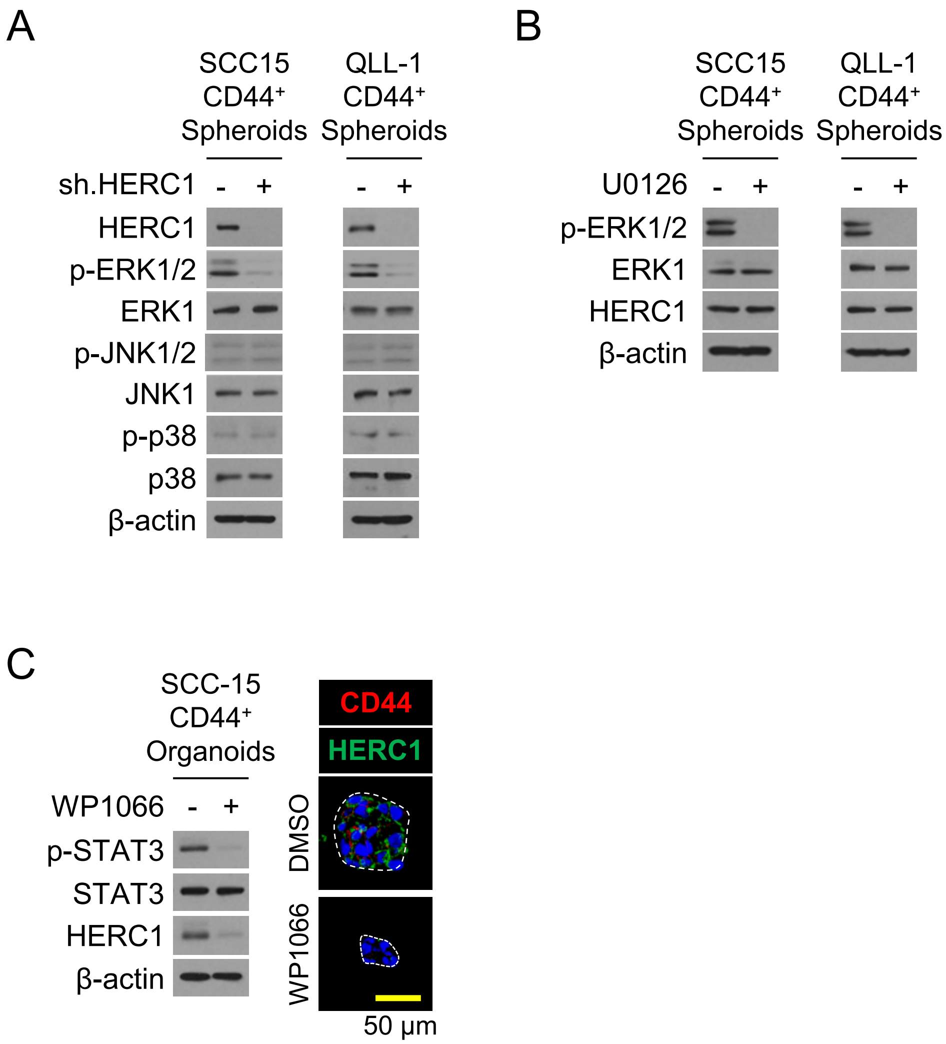

Supplementary Figure S7

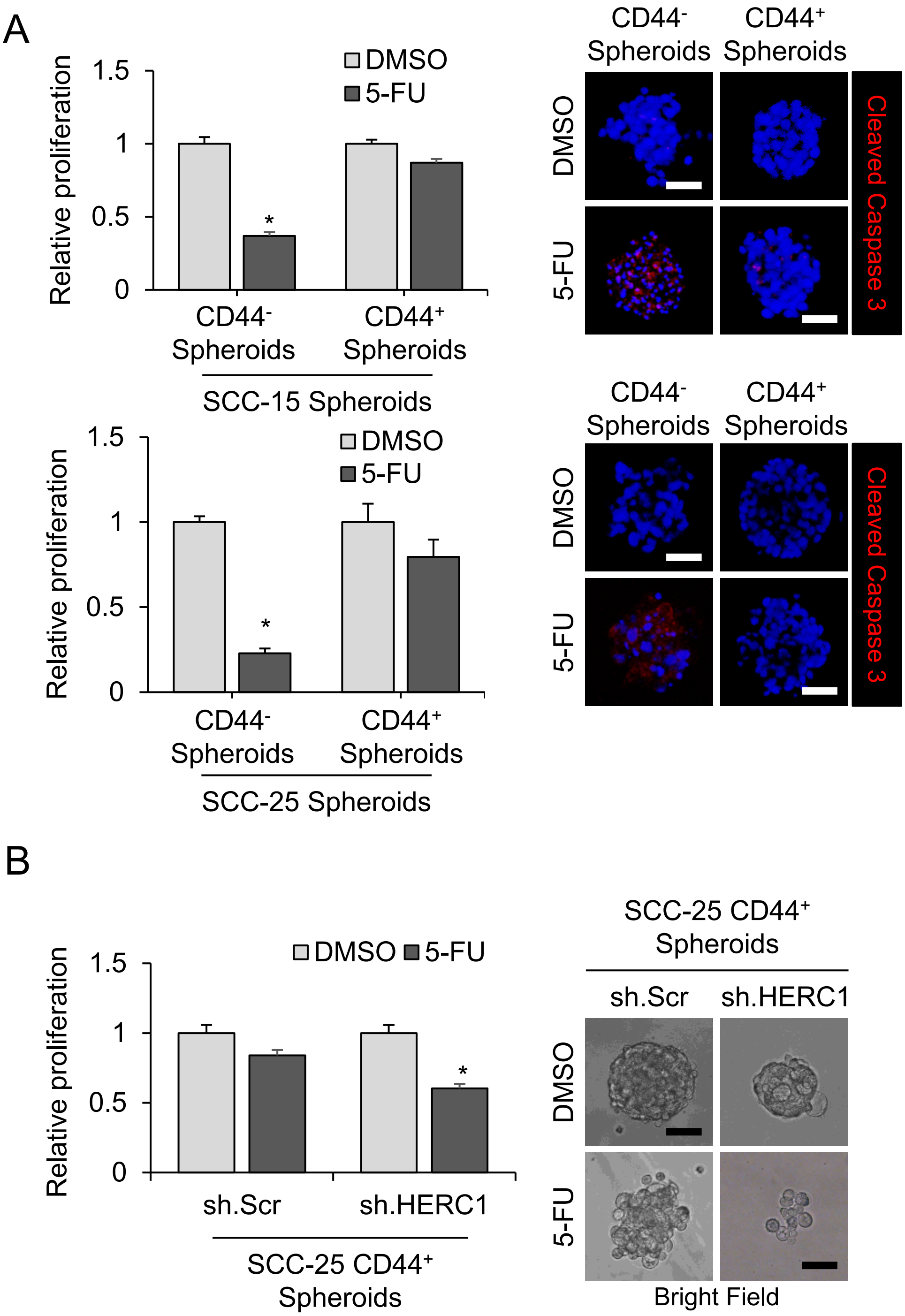

Supplementary Figure S8

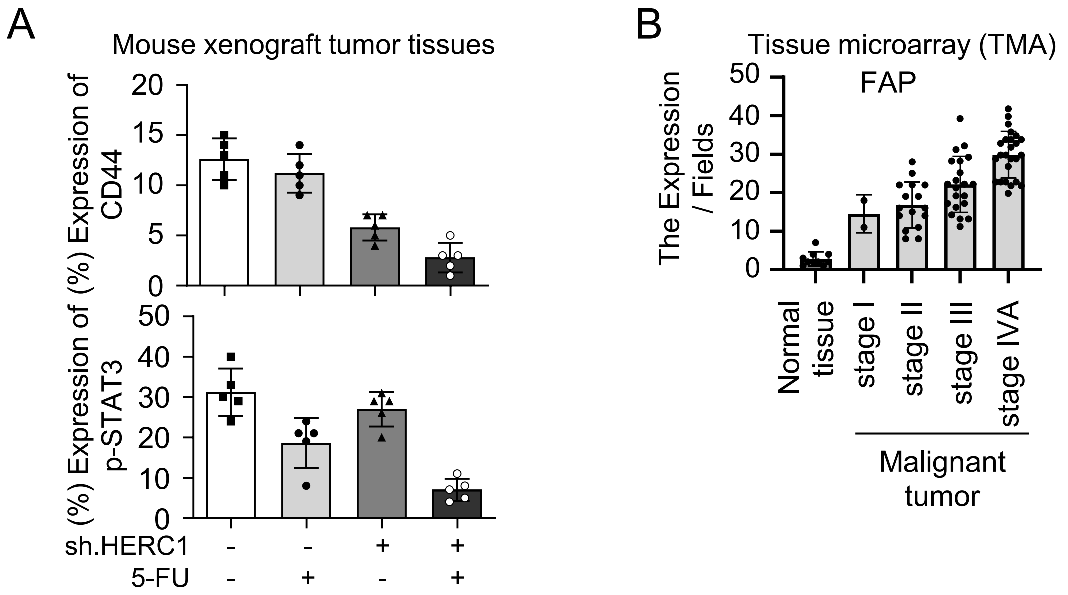

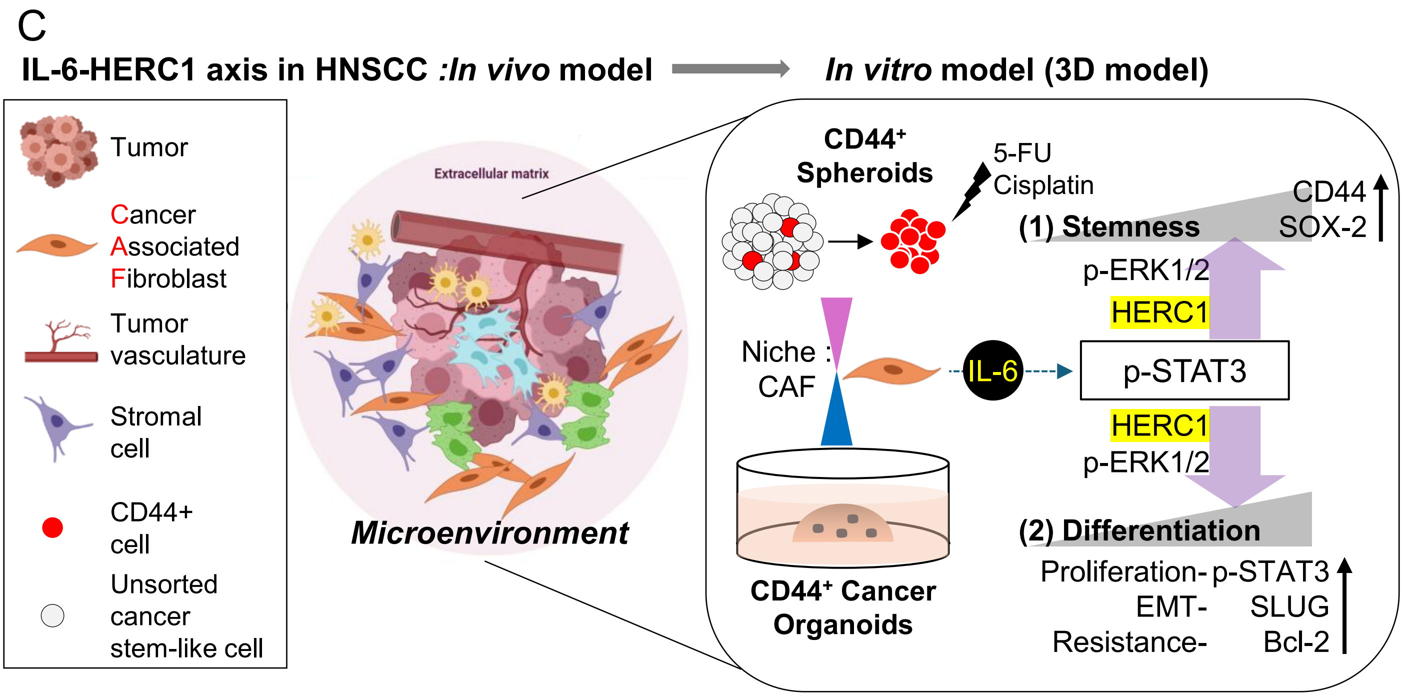

Supplement: Supplementary file 1 — Supplementary Material [file 41388_2026_3725_MOESM1_ESM.pdf]
